# A Computational Approach to Understand In Vitro Alveolar Morphogenesis

**DOI:** 10.1371/journal.pone.0004819

**Published:** 2009-03-13

**Authors:** Sean H. J. Kim, Wei Yu, Keith Mostov, Michael A. Matthay, C. Anthony Hunt

**Affiliations:** 1 UCSF/UC Berkeley Joint Graduate Group in Bioengineering, University of California, Berkeley, California, United States of America; 2 Department of Anatomy, University of California San Francisco, San Francisco, California, United States of America; 3 Cardiovascular Research Institute, University of California San Francisco, San Francisco, California, United States of America; 4 Department of Bioengineering and Therapeutic Sciences, University of California San Francisco, San Francisco, California, United States of America; Keio University, Japan

## Abstract

Primary human alveolar type II (AT II) epithelial cells maintained in Matrigel cultures form alveolar-like cysts (ALCs) using a cytogenesis mechanism that is different from that of other studied epithelial cell types: neither proliferation nor death is involved. During ALC formation, AT II cells engage simultaneously in fundamentally different, but not fully characterized activities. Mechanisms enabling these activities and the roles they play during different process stages are virtually unknown. Identifying, characterizing, and understanding the activities and mechanisms are essential to achieving deeper insight into this fundamental feature of morphogenesis. That deeper insight is needed to answer important questions. When and how does an AT cell choose to switch from one activity to another? Why does it choose one action rather than another? We report obtaining plausible answers using a rigorous, multi-attribute modeling and simulation approach that leveraged earlier efforts by using new, agent and object-oriented capabilities. We discovered a set of cell-level operating principles that enabled in silico cells to self-organize and generate systemic cystogenesis phenomena that are quantitatively indistinguishable from those observed in vitro. Success required that the cell components be quasi-autonomous. As simulation time advances, each in silico cell autonomously updates its environment information to reclassify its condition. It then uses the axiomatic operating principles to execute just one action for each possible condition. The quasi-autonomous actions of individual in silico cells were sufficient for developing stable cyst-like structures. The results strengthen in silico to in vitro mappings at three levels: mechanisms, behaviors, and operating principles, thereby achieving a degree of validation and enabling answering the questions posed. We suggest that the in silico operating principles presented may have a biological counterpart and that a semiquantitative mapping exists between in silico causal events and in vitro causal events.

## Introduction

Many internal organs in metazoa comprise liquid or gas filled cystic structures surrounded by a layer of epithelial cells. How such hollow structures are formed is a central problem in morphogenesis and tissue regeneration. Hollow structures are formed in vitro by a wide array of mechanisms [1], [2]. To better understand the mechanisms in an in vitro setting, epithelial cells have been cultured in 3D gels of extracellular matrix (ECM), such as collagen I or Matrigel®. When grown in 3D culture, Madin-Darby canine kidney (MDCK) or human mammary MCF10A cells can form similarly structured organoids, comprised of a monolayer of polarized epithelial cells with their apical surfaces facing a single central lumen. Proliferation and apoptosis are essential features of the process [3], [4]. When MDCK cells are maintained in 3D Matrigel cultures, polarity is efficiently achieved and coordinated with cell proliferation, enabling cystogenesis to occur by membrane separation without apoptosis [5]. In contrast, MCF10A cells utilize death of central cells to form hollow cysts [4]. Recently, Yu et al. [6] showed that primary human alveolar type II (AT II) epithelial cells maintained in 3D Matrigel cultures undergo cystogenesis to form alveolar-like cysts (ALCs) by a different mechanism which involves neither cell proliferation nor death. Cells within ALCs exhibit functions characteristic of their behavior in vivo, making that culture model appropriate for studying aspects of pulmonary alveolar development and function. Interestingly, in a repair-like process, ALCs form exclusively by cell aggregation followed by cluster expansion and remodeling, including hollowing (separation of cell membranes): there is no detectable cell proliferation or apoptosis. Taken together, the behaviors within these different cell culture systems demonstrate that different mechanisms (biological protocols) are being used to achieve the same stable structures. Identifying, characterizing, and understanding those mechanisms is essential to achieving deeper insight into this fundamental feature of morphogenesis.

However, the task is complicated by the fact that during cystogenesis, different cells can be simultaneously engaged in fundamentally different activities. For example, one AT II cell can be moving about within a cell cluster while another is migrating alone, and another is trying to attach itself to an early stage ALC, etc. No methods are currently available to comprehensively characterize either the variety of activities or their relative frequencies. Yet, the information is needed to answer such questions as these. When and how does an AT cell choose to switch from one activity to another? Why does it choose one action rather than another? Are several action options always available to each cell? Obtaining answers from in vitro studies is problematic because plausible answers are required in order to frame the hypotheses around which wet-lab experiments can be designed. Here, we report obtaining plausible answers using a rigorous, multi-attribute modeling and simulation approach that leveraged earlier efforts [7], [8], [9] using new, object-oriented, executable biology [10] capabilities.

**Figure 1:**
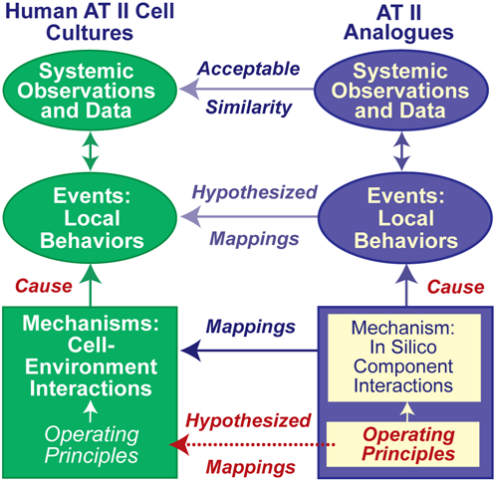
Relationships between in silico AT II analogues and AT II cells in culture. To distinguish simulation components and characteristics from in vitro counterparts, we use small caps when referring to the former. All systemic in vitro attributes are consequences of AT II cells interacting with each other and components of their environment. To create the analogue's mechanisms, we focused on the cell level, because cells are the system's primary functional units. We specified quasi-autonomous cell components that interact with all components in their adjacent environment. We required that each component map clearly to an in vitro counterpart. To enable remodeling of cell clusters into stable alveolar-like cysts, we used iterative analogue refinement to discover a set of axiomatic operating principles to which cells tightly adhered. The sum of local component interactions during execution represented the analogue's mechanisms. They gave rise to observable, measurable, systemic phenomena. A degree of validation was achieved when a set of in silico attributes achieved a prespecified Similarity Measure: i.e., measures of analogue attributes were within a prespecified range of corresponding measures of in vitro attributes. Upon validation, we could hypothesize that a semiquantitative mapping existed between in silico causal events and in vitro causal events. We could also hypothesize that the set of in silico operating principles had a biological counterpart.

The solution presented is based on the theory that when two *model* systems—in vitro AT II cultures and an in silico analogue—are composed of components for which similarities can be established, and the two systems exhibit multiple attributes that are similar, then there may also be similarities in the generative mechanisms responsible for those attributes. We demonstrate that for AT II cultures and the analogue described herein, those similarities exist. The approach and concept are diagrammed in Fig. 1. To make it successful, it was essential that the cell components of the culture analogue be quasi-autonomous.

We discovered a set of cell-level operating principles that enabled independent, interacting software objects, including those that mapped to individual AT II cells in vitro, to self-organize and generate systemic cystogenesis phenomena that were quantitatively indistinguishable from those reported in [6]. Component interactions during execution were the analogue's mechanisms. An initial, abstract analogue was iteratively improved so that an expanding set of the phenotypic attributes quantitatively matched data from AT II cultures. By so doing we strengthened in silico to in vitro mappings in stages at all three levels in Fig. 1, mechanisms, behaviors, and operating principles, and thereby achieved a degree of validation. Having accomplished that, we were able to answer the preceding questions for the AT II analogue. For example, at intervals, as time advances, each cell within analogue cultures uses updated information about its environment to classify its condition. The cell then selects and uses one of the axiomatic operating principles to specify and execute just one action for each possible precondition. Based on the results, we suggest that the in silico operating principles described herein may have a biological counterpart and that a semiquantitative mapping exists between in silico causal events and in vitro causal events.

## Methods

### Modeling approach and strategy

We used the synthetic or constructive approach [11], [12], enabled by agent-based modeling [13] and discrete event simulation [14], [15] methods described in Text S1. Fisher and Henzinger [10] called the approach executable biology. First, we specified the referent system, identified the perspective taken in the wet-lab and the system aspects on which to focus, and then stated the uses to which the model would be put. Next, we proposed building blocks and their functions, along with assembly methods so that the components and assembled system mapped logically to in vitro counterparts, as illustrated in Fig. 1. We refer to that system as an *analogue* to help distinguish this class of models from traditional, inductive models. Analogues were executed and measured in the same way as their referent. Data accumulated during executions were compared against data taken from the referent. When an analogue failed to achieve a prespecified Similarity Measure (SM), we revised it, validated it against its predecessor (cross-model validation) and then against referent attributes. When satisfied, a case could be made for each of the mappings in Fig. 1. The methods provide for establishment of plausible reductive hierarchies between mechanisms and phenomena by building more detailed and better-tuned analogues from a predecessor, in this case the one described in [7].

We first identified and rank-ordered a list of attributes characteristic of AT II cultures. The result is provided in Table 1. We divided the list into two parts: those to be targeted as part of this project, and those to be targeted as part of future projects. Including the latter was useful because it helped us avoid system design features that would likely require system reengineering in order for a descendent analogue to achieve one of those attributes. Next, we identified a subset of attributes to be targeted first. That initial subset was kept purposefully short to keep the fledgling analogue as simple as possible. Including unneeded complexity too early leads to prematurely complicated analogue designs. We targeted three attributes initially (Table 1). We sought the simplest components and mechanisms that would enable realizing those attributes. The mechanisms and system created was the foundational AT II analogue. The attributes excluded initially were ignored until validation against the initial subset of target attributes was achieved. The resulting foundational analogue was simplistic and somewhat unrealistic. It was improved iteratively using the following protocol: 1) select a new attribute from the list; 2) determine if the current analogue validates against the expanded target list, and if so, go back to step 1; 3) else, iteratively revise the analogue until the measured attributes are sufficiently similar to the expanded set of targeted attributes. Attributes were sufficiently similar when a prespecified, quantitative SM (discussed below) was achieved. We used a sequence of increasingly stringent SMs.

**Table 1:** Attributes targeted by AT II analogues.

Order targeted	Systemic attributes (primary human AT II cells; 3D Matrigel)
1	Single cells, acting autonomously, migrate and cohere upon contact
1	Cell clusters grow by aggregation only
1	Cells do not undergo proliferation or apoptosis
2	Cells in a cluster move collectively as a single structure
2	Cell clusters fuse to form larger bodies
3	Clustered cells shift their relative positions within the structure
3	Cell clusters develop into ALCs, each with a central lumen enclosed by a monolayer of composing cells
3	Larger ALCs form when the initial cell density increases
4	Cell aggregation is inhibited but cell speed maintained when β-integrin function (cell adhesion) is blocked
4	Cells moving slower in dense Matrigel form smaller clusters and thus smaller ALC
	**Future, targetable attributes**
	Apparent density-dependent upper limit to ALC size; ALC size range limited
	ALC lumen is filled with secreted fluid, lamellar bodies, and surfactant
	Dose dependent increase in ALC size following treatment with forskolin
	Increased fluid transport results in larger ALCs with distended lumens and cells stretched
	The ALCs are polarized with apical plasma membrane facing the lumen

### Specification of target attributes of in vitro AT II cell phenotype

The list of basic AT II cell attributes in Table 1 came from a recent study of primary human AT II cells [6]. The following were among the observations. When cultured in 2% Matrigel, primary AT II cells that were initially single or in small clumps aggregated. The cells were non-proliferating so cluster formation occurred by cell aggregation only. Clusters developed subsequently into alveolar-like cysts (ALCs), each comprising a central lumen devoid of cells enclosed completely by a monolayer of cells. The cells did not exhibit a significant level of apoptosis, so it was postulated that lumen formation occurred by hollowing: separation of cells within the cluster to create a hollow lumen. ALCs maintained a roundish shape without depressions or dimples. Their final diameter depended on initial cell density. When Matrigel concentration was increased from 2% to 10% to reduce the speed of cell movement, smaller cysts formed. To impair adhesion to other cells, AT II cultures were treated with a function-blocking antibody against integrin, a protein important for cell-cell and cell-matrix attachments. Cells so treated migrated normally, but formed smaller clusters that failed to aggregate further.

### Conceptual abstraction of the referent AT II cell culture system

We conceptually discretized AT II cell cultures into four components: cells, media containing Matrigel (matrix hereafter), matrix-free fluid (free or luminal space hereafter), and the space that contained them. We used objects to represent these components. To clearly distinguish simulation components and characteristics from in vitro counterparts, we use small caps when referring to the former. Matrix and luminal space were represented using passive objects. Cells were conceptualized as quasi-autonomous agents (as agents, they can schedule their own events and follow their own agenda). Time advanced discretely in simulation. The course unit of time was the simulation cycle, during which everything in the simulation had one opportunity to update. Within a simulation cycle, each cell, in pseudo-random order, was given an opportunity to interact with adjacent objects in its environment. Having objects update pseudo-randomly simulates the parallel operation of cells in culture and the nondeterminism fundamental to living systems, while building in a controllable degree of uncertainty. A mapping between simulation cycles and wet-lab time was specified toward the end of the validation protocol. Doing so too early limited and complicated future analogue improvements.

### System architecture and components

**Figure 2:**
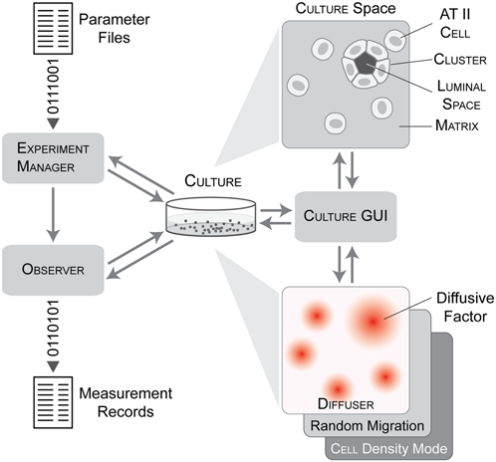
AT II analogue components and system architecture. The computational system consists of the core culture analogue, culture, and the framework components needed to support in silico experimentation and analysis. In vitro cell cultures and AT II analogue are both composite systems. A culture represents one in vitro cell culture. It is a composite of four object types: cells, matrix, free space, and cluster. A hexagonal grid provides the space within which the components interact. Cells are quasi-autonomous agents whose actions are driven by their internal logic (Fig. 3) and a set of axiomatic operating principles (Fig. 4). Cluster represents a coherent aggregate of two or more cells and can include free space. It has methods to manage group actions such as cluster migration. Matrix represents ECM and free space corresponds to aqueous material (e.g., ALC lumen) devoid of cells and matrix. Both are passive objects without their own logic. Cells and clusters can call on one of three migration modes: random, chemotaxis, and cell density-based. Diffuser is an extension to the culture space to simulate diffusion of an abstract factor called attractant that was used to guide chemotaxis. It uses a second hexagonal grid. Experiment manager is the experiment control agent. It prepares parameter files, manages execution of experiments, and processes experimental data for analysis and summary. Observer is a system-level module that automatically conducts and records measurements made on culture. Culture GUI provides a graphical interface to visualize and interactively probe culture during execution.

The model is a self-contained experimental system that comprises the core analogue and support components for experimentation and analysis (Fig. 2). Main system components are experiment manager, observer, culture, cell, cluster, matrix and free space. Additional components that extended functionality include culture graphical user interface (GUI) and diffuser. See Text S1 for full descriptions of the cluster, culture, and experiment manager components.

**Figure 3:**
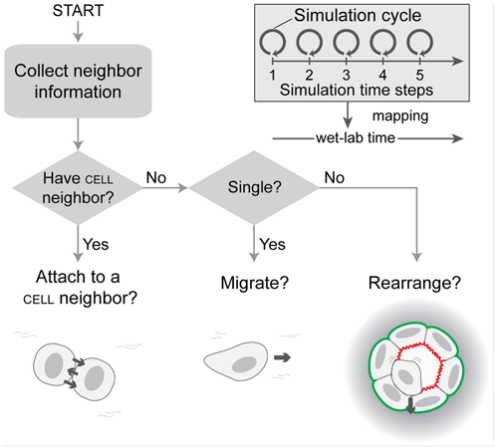
Encoded AT II cell step logic and the decision process. Simulation time advances in steps corresponding to simulation cycles. Each simulation cycle maps to an identical interval of wet-lab time. During a simulation cycle, every culture component is given an opportunity to update. Every cell, selected randomly, executes a step function each simulation cycle to decide what action to take based on its internal state (clustered or single) and the composition of its adjacent neighborhood. Enabled cell actions are cell-cell attachment, cell migration, and rearrangement within a cluster. With a specified probability, a cell in either state will attach to one of its neighboring cells. A single cell can migrate to an adjacent space. A cell within a cluster can rearrange with other cells composing the cluster. Cell and clusters are specified parametrically to migrate randomly, chemotactically, along a cell density gradient, or using a combination of random and directional movement.

**Figure 4:**
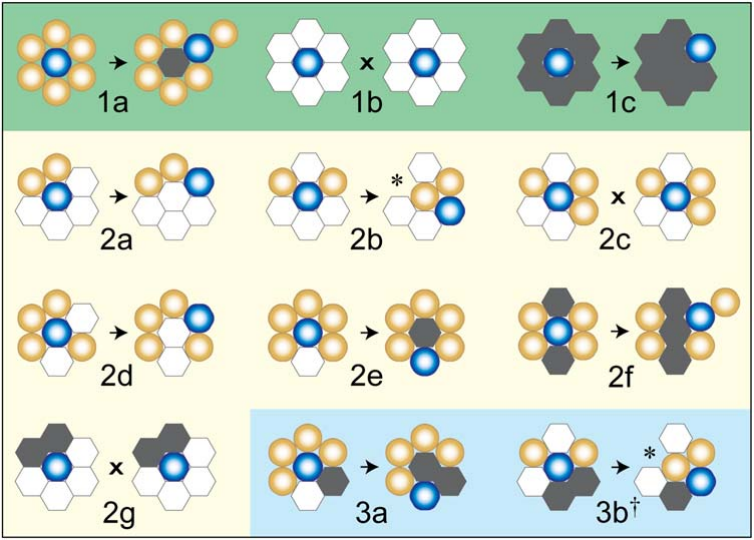
Cell axiomatic operating principles. A cell acts based on the composition and arrangement of its local environment. That environment can change from one simulation cycle to the next. Axioms are illustrated in three groups based on the variety of components adjacent to a center cell surrounded by six objects. We used observations reported in the literature, and those of AT II cell culture data [6], to encode, test, and iteratively refine candidate axioms to yield the final set, as described under Methods. Absent biological evidence, ones that moved the analogue closer to validation were selected over those that did not. For Axioms 1a–c, there is only one adjacent component type. For Axioms 2a–g, there are two, and for Axioms 3a–c, each of the three component types is present. The axiom precondition is on the left. On the right is the post-action configuration. Round, blue objects with white centers are the center cells executing the axiom. Round, gold objects having light centers are adjacent cells. White and gray hexagonal objects are matrix and free space, respectively. During each simulation cycle, each cell, selected randomly, has an opportunity to register the type and relative location of adjacent components. When a particular condition is met, there is one corresponding axiom-specified action (on the right). Axiom 1a: push a randomly selected adjacent cell (in the direction of that cell) and move to its location. Leave behind a free space. 1b: for this condition, do nothing (x). 1c: select an adjacent free space randomly and exchange places with it. Axioms 2a–2e apply when the adjacent space contains a mix of only cells and matrix. 2a: only one cell or a pair of adjacent cells: change places with either matrix adjacent to a cell. 2b: two nonadjacent cells: select randomly and then eliminate a matrix adjacent to either cell; move to its location and pull the other cell into the vacated center space. 2c: three adjacent cells (there are three such arrangements): do nothing (x). 2d: four adjacent cells: exchange places with either matrix. 2e: five adjacent cells: eliminate the matrix and move to its location. Leave behind a free space. 2f: the adjacent environment contains some mix of only cells and free space; push out a randomly selected cell neighbor and take its place. Leave behind a free space. 2g: the adjacent space contains some mix of only matrix and free space: do nothing (x). Axioms 3a–c: the adjacent environment contains all three objects types. 3a: only one matrix (there are nine possible configurations): eliminate the matrix and move to its location. Leave behind a free space. 3b: one adjacent cell is flanked by matrix (there are five possible configurations): select a free space randomly, eliminate it and move to its location. Pull the matrix-flanked-cell into the center location. ^†^ 3c: all other three-component arrangements: do nothing (x). * The object type adjacent to the pulled cell is pulled iteratively into its original location.

Discrete objects with eponymous names represent the essential cell culture components: cells, matrix, and free space. Cells have decision logic and axiomatic operating principles (Figs. 3 and 4) for interacting with the neighboring environment. Matrix and free space map to units of extracellular matrix and matrix-free material. Cells and surrounding objects have the same relative size. For simplicity, matrix maps to any cell-sized volume containing sufficient ECM to which AT II cells can attach. For this report, ECM refers collectively to the variety of components comprising 2% Matrigel culture media. Free space maps to similarly sized regions devoid of ECM and cells. Free space also represents luminal material and the material in pockets enclosed by cells. The latter are called luminal space when distinction from free space is useful.

A cluster is a cohesive aggregate of cells that can act quasi-autonomously, independent of individual cell activities. A cluster is created when two or more cells attach. Single cells that establish attachments to member cells are added to the cluster. Individual clusters that are adjacent and detected by member cells, can combine to form a larger aggregate. A cluster schedules its own events, which run at the same frequency of cell events. The cluster is deactivated and withdrawn when membership diminishes to one; the remaining cell reverts to single cell status. Each cluster uses an identical step function to determine its action. The step function is scheduled every simulation cycle. A cluster can either migrate a certain distance or do nothing. Migration speed and the probability of actual movement are specified parametrically.

A culture is an agent that maps to an arbitrary portion of the culture within one well of a multi-well culture plate. Culture uses 2D hexagonal grids to represent spaces in which cells, matrix, and free space objects are placed. Grids have toroidal topologies. For simplicity, each grid position is occupied by one object. That condition can be easily changed when the need arises. Visualization and user interaction are provided by a culture GUI. It extends the culture class with display and controller methods. Using the visual controller, the user can start a simulation or pause and access live states of culture grid positions and individual objects during simulation. Culture GUI also supports automatic recording of sequential images in different formats for post-simulation image generation.

A diffuser is a culture extension for simulating dispersion of a cell-created extracellular substance. A diffuser object is created only when chemotactic migration mode is enabled. A diffuser contains a grid and a step function to compute diffusion. The same hexagonal 2D grid type is used and aligned with the culture grid; however, the grid contains only numerical values. The culture start function initializes the diffuser with the specified initial attractant levels. The diffuser object is stepped and its diffusion algorithm executed a parameter-specified number of times within each simulation cycle. For example, the diffusion algorithm is executed 25 times during each simulation cycle when the parameter is set to 25. The algorithm provides a simple discrete approximation of diffusion based on parametrically defined diffusion and loss rates:
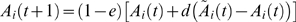
(1)where *d* and *e* are the diffusion and loss rates, *t* is the diffuser step counter, *A_i_*(*t*) is the substance level at grid position *i*, and *A* ~*_i_*(*t*) is the average attractant level across grid position *i* and its six neighboring locations. Maximum rates = 1. Attractant levels are capped at a maximum listed in culture specifications.

Experiment manager, the top-level system component, is an agent that provides experiment protocol functions and specifications. The agent controls, and has direct access to, observer, culture, and culture GUI. It can conduct experiments in default, visual, or batch mode. An experiment in default mode is simply a single execution. In visual mode, a culture GUI is created and the visualization console launched. Batch mode enables automatic construction and execution of multiple experiments, as well as processing and analysis of recorded measurements. A parameter file, such as that in Table 2, containing requisite values is accessed to prepare culture and perform an experiment. After completion of all experiments, basic analytic operations are used to collect and summarize experimental data.

**Table 2:** Key AT II analogue parameters.

Component	Parameter	Base value	Description
Experiment manager	*MonteCarloRun*	100	Number of Monte Carlo runs per experiment
	*SimTimeSteps*	100	Simulation cycles executed per run
Culture	*GridWidth*	100	Culture grid width
	*GridHeight*	100	Culture grid height
	*InitCellPop*	3000	Initial cell population
	*MoveMode*	0*	Cell/cluster migration mode
Diffuser	*StepMultiples*	25	Diffuser iteration per simulation cycle
	*DiffusionRate*	0.4	Solute diffusion rate
	*EvapRate*	0.05	Solute evaporation rate
	*MaxSoluteLevel*	50,000	Maximum solute concentration per grid unit
Cell	*MaxPushNum*	5	Push iteration limit
	*MaxPullNum*	5	Pull iteration limit
	*CellAdhProbSingl*	0.2	Cell-cell attachment probability (single)
	*CellAdhProbClust*	0.01	Cell-cell attachment probability (clustered)
	*CellMoveSpd*	1	Grid unit distance moved per time step
	*MinSoluteProd*	3000	Minimum solute production level
	*MaxSoluteProd*	8000	Maximum solute production level
	*NeighbChckRadius*	5	Local cell density radius
	*SigmoidShift*	12	Culture grid exit control function shift
	*SigmoidScale*	2	Culture grid exit control function scale
	*MinActivCellNum*	6	Minimum cell number to enable grid exit
Cluster	*ClustMoveSpd*	1	Grid unit distance moved per time step
	*MovSigShift*	5	Cluster movement control function shift
	*MovSigScale*	2	Cluster movement control function scale

* 0, random migration; 1, chemotaxis; 2, cell density-based migration.

Observer is responsible primarily for recording measurements. It is created and assigned to a culture when initialized. Observer creation is a system option selected only when detailed measurements are needed. Observer is stepped and its probe method called at the end of every culture simulation cycle. The probe method scans the culture internals and performs measurements. Measured culture attributes include total cell population, cumulative migration distances of individual cells, occurrences of cell-cell attachments, cell activities in terms of axiom usage, and the number of multicellular structures formed and their sizes. These measures are recorded as time series vectors; the data are written to summary files at simulation's end.

### Designing AT II cell analogues

Cells use the axiomatic operating principles and protocols illustrated in Figs. 3 and 4 (discussed in detail below). They have a basic set of member variables and access methods that, when needed, can be extended for representation of more specific cell types. Each AT II cell has the same step function in which an environment assessment and a call for an appropriate action are made. The step function is scheduled each simulation cycle. To achieve the initial set of target attributes, we defined what we judged to be a minimal set of actions (Fig. 3): migrate, attach to an adjacent cell, and rearrange within a cluster. Cells are capable of three types of migration separately or in pairs: random movement, chemotaxis, and cell density-based migration. A cell can switch its migration mode during execution, for example from random movement to chemotaxis when the chemotactic mode is enabled and a gradient of attractant is detected. Migration speed is adjustable. Moving one-cell-width per simulation cycle maps to moving ∼6.8 µm/h in vitro. The cell-cell attachment action is executed when two cells are in physical contact, and the probability of actual attachment can be changed parametrically. Within a cluster, cells can move or rearrange. Cell rearrangements within a cluster are specified using the axiomatic operating principles illustrated in Fig. 4 and discussed below. A cell selects just one axiom and corresponding action during each simulation cycle. Positional rearrangement of a cell in a cluster can involve iteratively pushing or pulling neighboring cells. Every cell maintains a state variable to denote whether it is a member of a cluster. Cell detachment can occur during rearrangement; a detached cell reverts to the non-clustered state. Additional details on the individual cell actions are provided in Text S1.

### Challenging in silico predictions in vitro and testing mechanistic hypotheses in silico

Cell rearrangement activities are specified using axioms. Use of the term axiom reinforces that our computational model is a mathematical, formal system and that analogue execution is a form of deduction from the axioms or assumptions explicitly programmed into the model. An axiom specifies a precondition and corresponding action. Preconditions are defined in terms of neighboring object configurations. During update, clustered cells execute one of two operations: relocate or remain in place. Experimental observations [6] precluded other basic processes, such as proliferation and death. Every precondition was assigned an action: one of those two operations.

We used observations reported in the literature to help avoid axioms that may have been judged abiotic and to prefer axioms for which supporting evidence was available. Absent evidence, variations of an axiom were implemented and the consequences (in silico predictions) observed upon execution. We then searched the time-lapse movies of experiments [6] for supportive or falsifying evidence. Having the movies meant that we did not need to undertake new wet-lab experiments to test critical in silico predictions. When candidate axioms were rejected, we revisited the movies focusing on the somewhat different aspects of the recorded phenomena that were brought into focus by failed predictions and actions; those aspects may have escaped notice during earlier viewing (when the phenomenal aspect on which we focused was different). So doing allowed us to induce somewhat new mechanistic hypotheses that we then strove to encode in a revised set of axioms. Execution of each revised analogue tested the revised mechanistic hypotheses and provided a new set of phenomena, closing one iterative refinement cycle. That process of iterative instantiation, rejection (or acceptance), and revision of axioms along with concurrent revision of the preconditions moved the analogue closer to validation. That procedure yielded the axioms illustrated and described in Fig. 4. Their preconditions refer to the composition of six neighboring objects. During simulation, Axioms 1a, 2a, b, e, and f are preempted when a decision-making cell chooses to move off the culture grid. The preemption frequency is specified using a threshold function and parameters. The action was needed to more closely simulate observed 3D phenomena. Iterative steps taken for developing individual axioms and the quasi-3D simulation method along with more detailed descriptions of axioms are provided in Text S1.

### Similarity Measures used for model validation and refinement

Our plan was to discover and invent cell variants that could survive (or not) increasingly stringent similarity demands. Our initial, least stringent Similarity Measure, SM-1 was that >50% of clusters having ≥6 cells develop into ALCs. At each stage, several analogues, exhibiting one or more differences (in components and their logic, for example), competed to achieve the targeted SM. An analogue was falsified and discarded when no parameterization was found that enabled it to achieve the targeted SM. When all analogues were falsified, we went back to the drawing board. Once SM-1 was achieved, we enforced a stricter SM, SM-2, reflecting an expanded set of phenotypic attributes, and iteratively revised the analogue and cell axioms until >98% of clusters developed into ALCs. The third SM, SM-3, required that SM-2 be achieved and that ALCs have a mean shape value (*S(c)* defined below) <0.6. To do so, we used a simple shape analysis algorithm defined below. It computes a numerical cyst shape score. SM-4 increased stringency further; it required achieving a mean shape value of <0.3 (smaller is preferred). SM-5 increased stringency further; it required that SM-4 be achieved and that mean, simulated ALC diameters be within 10% of in vitro values. Achieving SM-5 was challenging and required iterative refinements, so we set two intermediate milestones: that SM-4 be achieved and that mean, simulated ALC diameters first be within 50% of in vitro values, and then within 25% of in vitro values. After iterative refinement and parameter tuning, separate analogues, each using just one of the three cell migration modes (random, chemotactic, cell density-based, discussed below) achieved the 50% and later the 25% milestones. Analogues using just the random cell migration mode were falsified: none achieve SM-5. Analogues using chemotactic or cell density-based migration modes that could achieve SM-5 were identified. That motivated setting an even more stringent SM, SM-6. It required that SM-5 be achieved and that mean, simulated ALC diameters be within 2% of in vitro values. SM-6 was achieved by an analogue that used the cell density-based migration mode along with some random movement.

The cyst shape analysis algorithm computes shape score, *S*, of a cyst, *c*, based on the ratio of the area (*A_c_*) enclosed by *c* to the hexagonal area (*A̅* *_c_*) enclosed by an ellipse circumscribing *c* as follows:
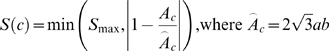
(2)
*S_max_* is the maximum possible score and has been calibrated to 1.0; *a* and *b* are semimajor (one half the major) and semiminor (one half the minor) axes of the enclosing ellipse. The major axis of the ellipse corresponds to either the length (i.e., the maximum horizontal span) or height (i.e., the maximum vertical span) of *c*, whichever is greater; the lesser becomes the minor axis. Lower *S(c)* values are preferred. Simulated structures with nonconvex or irregular shapes are assigned high scores, while convex contours generally are given scores closer to zero.

### Mapping of measurement units

Mappings from measurements taken on epithelial cell cultures to corresponding measurements taken in vivo are rarely 1∶1. They are often nonlinear and feature dependant. To establish a quantitative mapping, a mapping model is needed, necessitating specifying several assumptions. To enable a similar relationship between analogues and AT II cell cultures, we separated the mapping from analogue to referent from the actual analogue itself, and made the analogue fully relational. An advantage of doing so is that, as new wet-lab data becomes available, adjustments can be made to the mapping model without having to reengineer the analogue. Based on measurements of AT II cell migration, we devised a provisional mapping of analogue measurement units to in vitro metric units to enable direct comparison of simulation and in vitro data. Cells in culture swell, shrink, become more compact, and stretch as they form multicellular structures. Nevertheless, to specify the mapping, we assumed that average AT II cell shapes and sizes were relatively constant. In the current analogue, one grid unit corresponds to one cell-width. In vitro, their average diameter is ∼8.5 µm, so on average, the width of one grid unit maps to 8.5 µm. To map the simulated time to wet-lab time, we relied on the finding that AT II cells in 2% Matrigel cultures migrate ∼1.7 µm in 15 minutes [6]. Within simulations, cells using the parameter values in Table 2 move one grid unit per simulation cycle, and one simulation cycle maps to ∼88 minutes. The analogue and in vitro experimental measures are discussed further in Text S1.

### Simulating AT II cell culture experiments

To the extent possible, design of simulation experiments followed the experimental design and protocols detailed in [6]. The following represent a standard cell culture design and execution. First, the top-level system component, experiment manager, was instantiated and initialized using default parameter values or those from a parameter file, if specified. The experiment manager instantiated a new culture with specified or default grid dimensions; the new culture's grid was filled completely with matrix. The culture grid represents the observed *xy*-plane of an AT II cell culture. An observer was instantiated and its step added to the event schedule, so measurements could be made and recorded during simulation. A diffuser was added if chemotactic migration was specified. Next, cells were initialized and randomly distributed on the culture's grid, overwriting resident matrix objects. The initial cell population was specified parametrically; it mapped to the initial cell density of in vitro culture. As cells were initialized, their steps were added to the event schedule. Initially, every cell was in a non-clustered state. Simulation started when the initialization of culture grid contents was completed. After simulation, the recorded measurements were written to files and the culture was destroyed. A new culture was created for each repetition when the experiment manager was executing in batch mode.

We conducted standard cell culture experiments having initial populations of 100 to 6,000 cells in 100 cell increments. Culture width and height were set to 100, unless otherwise noted. Results from use of larger grid sizes were explored: they did not produce noticeably different outcomes. Each simulation experiment comprised 100 Monte Carlo (MC) runs. We collectively executed 60 independent experiments executed for 100 simulation cycles.

We investigated behavioral and phenotypic consequences of altering cell migration mode, cell-cell attachment probabilities, cell migration speed, and the axioms governing clustered cell actions. Changes in migration mode and speed, and attachment probabilities were made by changing corresponding parameter values. Alteration of the axioms required modification of the analogue implementation. Aside from these changes, the standard experiment design and execution protocols were followed. See Text S1 for detailed descriptions of the experimental design of AT II cultures having altered properties.

### Implementation tools

The model framework was implemented using MASON Version 11. MASON (http://cs.gmu.edu/~eclab/projects/mason) is a discrete-event, multi-agent simulation library coded in Java. We used R 2.5.1 (http://www.r-project.org) and Microsoft Office Excel 2003 (http://office.microsoft.com/excel) for statistical analysis of simulation data and generation of data figures. Batch simulation experiments were performed on a small-scale Beowulf cluster system consisting of one master node and seven client nodes, each equipped with a single Intel Pentium 4 3.0-GHz CPU and a 2-GB SDRAM memory. For model development, testing, analysis, simulation image processing, and video production, we used personal computers. Computer codes and project files are available at <http://biosystems.ucsf.edu/research_epimorph.html>.

## Results

The targeted AT II cell attributes are listed in Table 1. We begin by describing AT II analogues that validated by achieving the most stringent Similarity Measure, SM-6. Cells used two modes of movement. We also describe the behaviors of three analogues that were otherwise the same, except that cells used only one migration mode. We conclude with descriptions of how changes in the values of two sets of important parameters influenced analogue attributes: one parameter set controlling cell-cell attachment probabilities and another specifying cell and cluster movement within cultures.

### AT II analogues and their in vitro counterparts exhibit multi-attribute similarities

An analogue design objective was that simulation results exhibit specific characteristics that, when measured, would be quantitatively similar to corresponding measures of AT cultures. AT II analogues were abstract, working models of interacting, quasi-autonomous components. They were *not* intended to be mathematically precise descriptions of mechanisms thought to be used by AT II cells in vitro. The usefulness of an AT II analogue is judged in part by how well it mimics the attributes targeted.

Proliferation was not allowed because none was detected in vitro. For the same reason, cell death was not an option. However, cell division and death options can be added when it becomes necessary to validate for corresponding in vitro evidence. At the targeted level of resolution, that could be done using methods similar to those developed earlier [7], which used simple cloning and self deletion to mimic cell division and death, respectively. On the other hand, defining conditions under which either action takes place is not straightforward. As further insight from wet-lab studies becomes available we can proceed with the iterative refinement protocol described in Methods to expand the current set of axioms to include cell division and death options.

**Figure 5:**
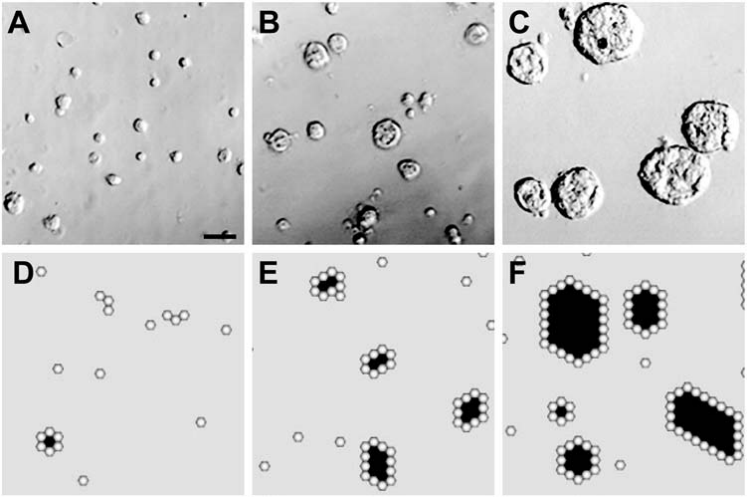
ALC growth in simulated and in vitro AT II cell cultures. (A–C) Phase-contrast pictures after 4 d in 2% Matrigel. (A) Cells were plated at 1×10^4^ cells/cm^2^; (B) plated at 5×10^4^ cells/cm^2^; (C) plated at 25×10^4^ cells/cm^2^. ALCs were roundish without obvious depressions or dimples. Final ALC sizes were dependent on initial cell density. Bar: ∼50 µm. (D–F) Shown are images of simulated culture, after 100 simulation cycles (∼6.1 days in vitro), starting with AT II analogues parameterized as in Table 2. Note that a hexagonal cyst within the discretized hexagonal space maps to a roundish cross-section through an ALC in vitro. Objects with white centers are cells. Gray and black spaces represent matrix and free (or luminal) space, respectively. Each simulated ALC in (D–F) maps to cross-sections through corresponding roundish ALCs in cultures. A regular, hexagonal ALC in hexagonal space maps to a circular cross-section in continuous space. The initial cell populations were (D) 200 cells (which maps to ∼1×10^4^ cells/cm^2^ in vitro), (E) 1,000 cells, and (F) 5,000 cells seeded randomly across the culture space. As in vitro, larger ALCs formed when the initial cell population was larger.

**Figure 6:**
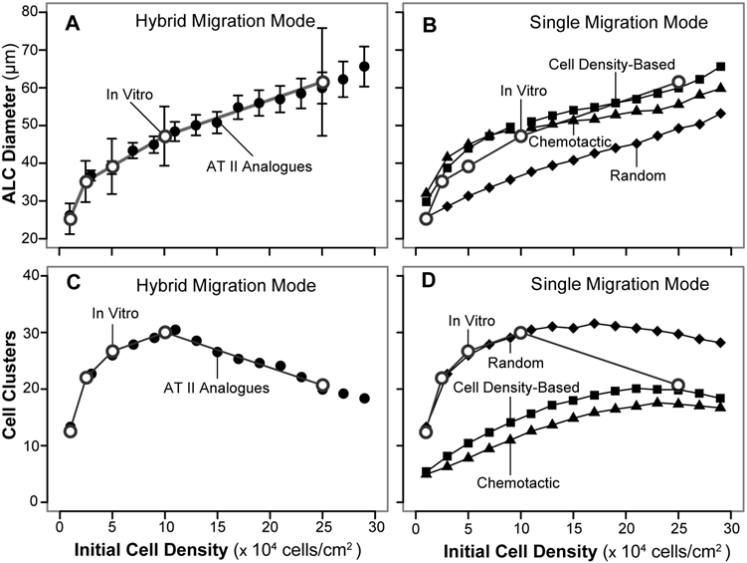
AT II analogues and AT II cell cultures can exhibit quantitatively similar, phenotypic attributes. (A) Mean ALC diameters for both systems are graphed as a function of initial cell density. Open circles: mean in vitro diameter after 5.7 days; vertical bars: ±1 S.D. for 25 observations. Filled circles: mean analogue diameters after 100 simulation cycles (∼6.1 days) using the AT II analogues that achieved the most stringent Similarity Measure (SM-6); bars: ±1 S.D. for 100 simulations. The dominant migration mode used by all AT II analogues was cell density-based. At initial densities of ≤2,000 cells (maps to ≤10^5^ cells/cm^2^ in vitro), 10–15% of movement events were specified randomly to be random moves rather than cell density-based. At higher initial densities, all movement events used the cell density-based mode. (B) Open circles: same as in (A). Filled symbols: mean results as in (A), except that all migration events used either cell density-based mode (squares), the chemotactic mode (triangles), or the random migration mode (diamonds). Results using the cell density-based and chemotactic modes achieved SM-5, whereas the analogue using the random migration mode failed to do so. (C) Open circles: final, mean cluster count (averaged over three culture wells) for the in vitro experiments in (A) graphed vs initial plating density. Filled circles: final mean cluster count for the AT II analogue experiments in (A). The two sets of results are experimentally indistinguishable. (D) Open circles: same as in (C). Filled symbols: mean results as in (C) except that all migration events used one of the indicated modes.

Following the logic diagrammed in Fig. 3, and using the axioms in Fig. 4, cells parameterized as in Table 2 produced culture level behaviors that qualitatively and quantitatively matched those observed in vitro. Examples are provided in Fig. 5. Detailed results are graphed in Figs. 6–8. Migrating single cells formed cell-cell attachments, which led to formation of small aggregates. Some aggregates migrated and merged with cells and other aggregates to form larger clusters. Cells within clusters rearranged themselves into configurations dictated by the axioms, causing adequately sized clusters to develop progressively into alveolar-like cysts (ALCs) having a luminal space surrounded by a cell monolayer (Videos S1, S2, S3). ALCs maintained convexity and had no dimples; most remained stable until the simulation terminated. Note that a structure having a regular hexagonal shape in hexagonal grid space (used by all cultures) maps to a circle in continuous space.

**Figure 7 pone-0004819-g007:**
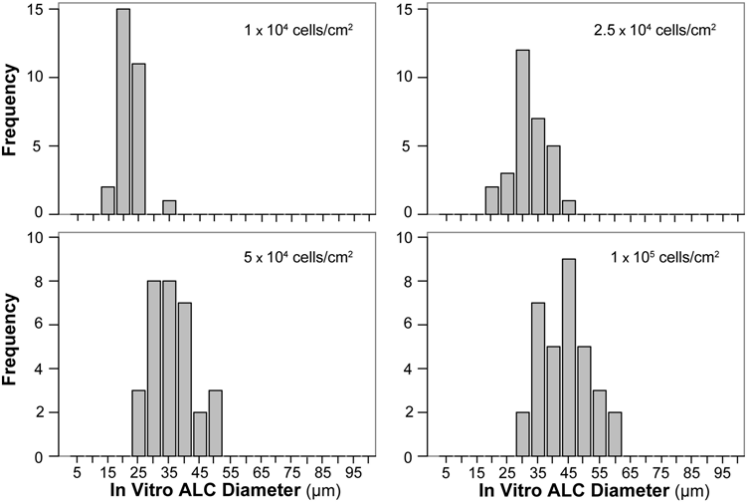
ALC size distributions in vitro. ALC frequencies within 5 µm intervals were measured after 5.7 days in vitro. The experiments are the same as those in Fig. 6. Frequency distribution characteristics depended on initial cell density. The distribution and median size shifted to larger diameters as initial cell density increased. In addition, the smallest ALCs became larger as initial cell density increased. Distribution tails in the direction of larger sizes appeared to be truncated.

**Figure 8:**
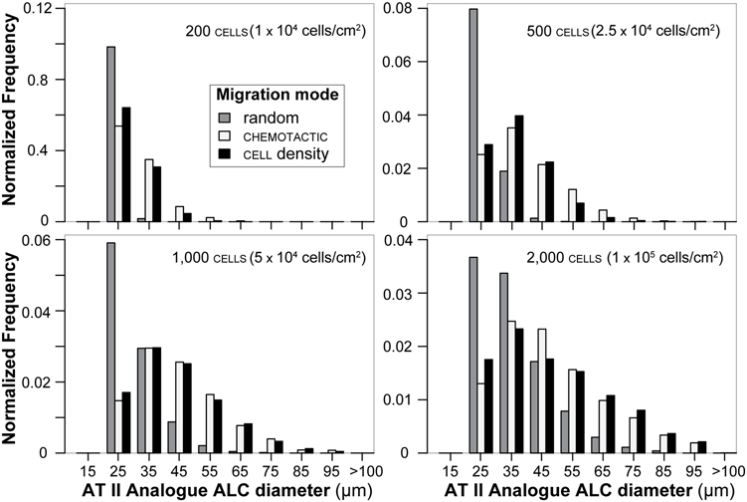
ALC size distributions for AT II analogues. Analogue ALC frequencies within 5 µm intervals were measured after 100 simulation cycles (maps to ∼6.1 days in vitro) for each of the three migration modes. The frequencies were normalized to fraction of total count for each migration mode and then averaged over 100 analogue cultures. The experiments are the same as those in Fig. 6B, D. Frequency distribution characteristics depended on initial cell density and migration mode. Initial cell densities are listed along with the corresponding in vitro density to which each mapped (in parentheses). Distribution tails in the direction of larger sizes were not truncated, as appeared to be the case in Fig. 7.

In vitro, the average ALC size increased monotonically with initial cell density. We observed the same during simulations: mean values and their standard deviations are graphed in Fig. 6A. A cell's width maps to ∼8.5 µm. In sparse cultures that started with 200 cells, which mapped to ∼1×10^4^ cells/cm^2^ in vitro, cells formed small ALCs that averaged 25 µm in diameter, essentially the same as the referent mean diameter (Fig. 5A and D; Fig. 6A). In denser cultures that started with 1,000 cells (5×10^4^ cells/cm^2^ in vitro), ALC diameters averaged 38 µm, similar to the average 39-µm diameter observed in vitro (Fig. 5B and E; Fig. 6A). At the highest density of 5,000 cells, which maps to 25×10^4^ cells/cm^2^ in vitro, cells produced ALCs that averaged 59 µm in diameter, only slightly smaller than the average 62-µm diameter observed in vitro (Fig. 5C and F). We also counted the number of clusters both in vitro and in simulations (Fig. 6C); the pattern, as a function of initial cell density, was the same for both.

### Three cell migration modes were explored

Random migration of both AT II cells and clusters thereof is the simplest movement mechanism to simulate. However, inspection of the time-lapse in vitro movies (Videos S4, S5, S6) makes clear that other mechanisms were operative and may dominate ALC formation. For example, there are instances of AT II cells and small clusters gravitating towards apparent rendezvous points (e.g., Video S4, ∼66 h, lower-left; Video S5, ∼23 h, upper-left). To explore plausible, alternative mechanisms, we gave cells two additional movement modes: chemotaxis and cell density-based movement. In chemotactic mode, both cells and clusters sensed differences in the levels of a diffusing, cell-produced attractant in their local environment, and moved to a neighboring location that had the highest attractant level. Cell density-based migration was based on observational evidence that cells and clusters are both drawn towards the most densely populated region of a cell's local environment.

When cells and clusters, parameterized as in Table 2, used only random migration, the analogues failed to achieve SM-5. However, those that used the chemotactic or cell density-based mode (Fig. 6B) produced ALC diameters that were adequate to achieve SM-5. The cell density-based mode produced outcomes that were visually closer to in vitro observations, but the results failed to achieve SM-6. We documented that cells using chemotaxis or the cell density-based mode produced aggregation patterns that resembled those of AT II cells in vitro (Videos S7, S8): cells and early clusters tended to move towards common points in a convergent manner similar to that observed for AT II cells.

Interestingly, when attention was shifted to cluster count, regardless of size or ALC status, we observed that cluster counts obtained using both the chemotactic and cell density-based movement modes, at low and intermediate initial densities, were distant from corresponding in vitro values (Fig. 6D). That discrepancy was noteworthy especially given that cluster sizes formed at lower initial densities using only the random migration mode were similar to measured in vitro values. We took these observations as evidence that in vitro, AT II cells and clusters likely used more than one migration mode. The analogues in Fig. 6A and C achieved SM-6 by using both random and cell density-based migration modes: at initial densities of ≤2,000 cells (≤10^5^ cells/cm^2^ in vitro), 10–15% of movement events were specified randomly to use the random migration mode. Otherwise, they used the cell density-based migration mode. At higher initial densities, all movement events used the cell density-based mode.

The frequency distribution of in vitro ALC diameters was dependent on initial cell density (Fig. 7). The distribution peak and size of the smallest ALC increased with increasing initial density. The distributions also appeared truncated: the largest diameters were about 2–2.5 times the smallest, suggesting a mechanism that may preclude formation of extra-large ALCs. The frequency distributions of ALCs from analogues that used a single migration mode were clearly different (Fig. 8). When using random movement mode, most ALCs were the smallest size formed, independent of initial cell density. For both chemotactic and cell density-based movement modes, peak diameter was larger than the smallest size and was shifted to larger values with increasing initial cell density. The size range also increased with increasing initial cell density.

### Relative axiom use reflected changes in each cell's local environment

**Figure 9:**
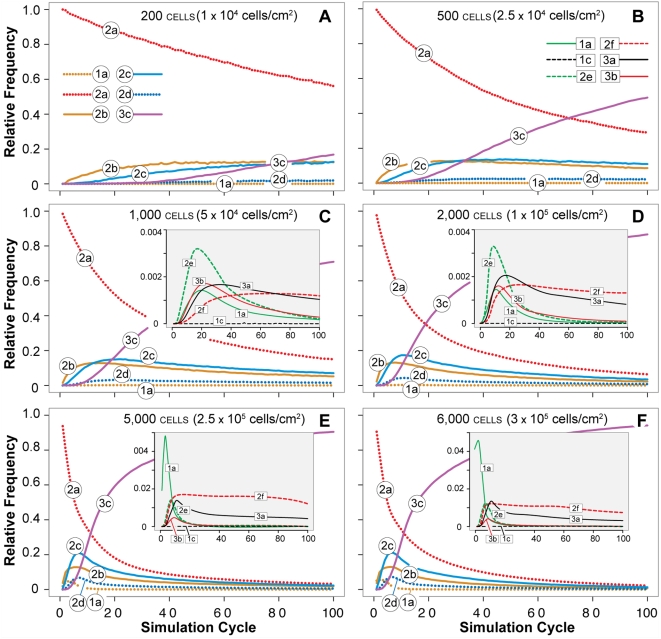
Axiom usage. Frequency of axiom usage is plotted versus simulation cycle for the AT II analogue as in Fig. 6A. Ten simulation cycles map to ∼14.6 hours. Relative axiom use depended on initial cell densities, which are listed along with the corresponding in vitro density to which each mapped (in parentheses). The variance in use frequency across simulation cycles for the less frequently used axioms was large. In the inserts, trend lines were used to make patterns more evident. Early in simulations, Axioms 2a, 2b, and 2c were used most frequently as cells rearranged themselves and condensed into packed clusters. Axioms 1a and 2e were executed most often early in ALC development to provide for luminal space creation. As simulations progressed and ALCs matured, Axiom 3c (do nothing) was executed more frequently: stable structures were forming and for most cells, no further rearrangement was needed. (A–C) At low-to-moderate cell densities, Axioms 2b and 2c also applied often when cell clusters were unable to grow further and develop into ALCs. The remaining axioms showed only limited usage (insets), yet they were essential in achieving targeted attributes. For example, Axiom 1c was essential in enabling cells trapped within lumen spaces to merge with its parent cluster. (D–F) In densely populated cultures, usage of Axioms 2e, 2f, 3a, and 3b increased several fold (insets), especially early in simulation. ALCs developed and matured sooner in dense cultures as indicated by the earlier increases in Axiom 3c usage.

Individual axiom usage during the simulation experiments graphed in Fig. 6A are provided in Fig. 9. Axioms 2a and 3c were used most frequently, followed by Axioms 2b and 2c. Cells executing Axiom 2a, 2b, and 2c had only cell and matrix neighbors; they were members of developing clusters that did not yet have a complete luminal space. Cells in maturing ALCs executed Axiom 3c, so not surprisingly that axiom was executed most frequently as the simulation progressed. The remaining axioms, although needed, exhibited infrequent use.

Usage patterns changed dynamically over time reflective of the changes in a cell's extracellular composition and arrangement. Although relative axiom use patterns were qualitatively similar for all initial cell densities, the specific details were both simulation cycle and initial cell density dependent. Cells in sparse cultures exhibited more frequent and extended use of Axioms 2b and 2c, whereas in densely populated cultures, use frequencies of Axioms 2e, 2f, 3a, and 3b increased several fold. The infrequently used axioms were, nevertheless, critical to the formation of morphologically normal ALCs. They were used mostly in the early phase of ALC development. Blocking execution of each of these axioms disrupted normal ALC growth (data not shown). Specifically, blockage of Axiom 1c or 2f led to frequent appearance of isolated clumps of cells within ALC lumens. When executions of Axioms 1a and 2e were blocked, cell aggregates failed to develop a luminal space and no ALCs formed. Without Axiom 3a, the cells formed ALCs having irregular, nonconvex shapes.

### The consequences of altering cell-cell attachment probabilities matched in vitro observations

Cell-cell adhesion is a basic requirement for development of multicellular structures. In [6], blockage of β1-integrin inhibited cell-cell adhesion but not cell movement. Some clusters formed but not ALCs. Cells in large clusters rearranged often without developing into stereotypical spherical structures, whereas some small clusters did stabilize into round structures, but without lumens. By making two adjustments, we observed essentially the same behavior (not shown) using the cells from Fig. 6A and C. We reduced the cell- cell attachment probability toward zero, but not to zero; within analogues, adhesion and attachment were synonymous. We also reduced the frequency of following the actions required when the preconditions of Axioms 1a and/or 2e were met, from 100% to 50% or less.

To explore further the consequences of changing the cell-cell attachment probability, we conducted a series of simulation experiments to answer the following question. Would increasing attachment probability above 0.2 (Table 2) for the cells from Fig. 6B and D under some or all initial density conditions improve outcomes sufficiently so that SM-6 could be achieved using just one movement mode rather than the two used to achieve the results in Fig. 6A, C?

**Figure 10:**
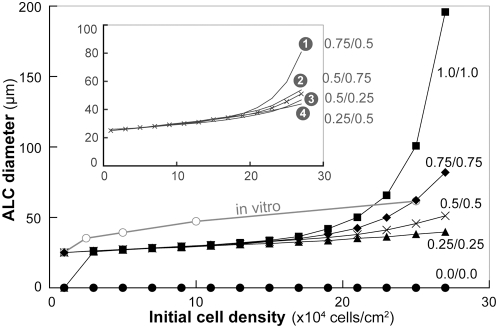
Final ALC diameter following changes in cell-cell attachment probabilities. For simplicity, experiments were conducted using the random migration mode, as in Fig. 6B. Upon contact, cells formed intercellular attachments; attachment probability was specified parametrically (Table 2). One parameter controlled the attachment probability of clustered cells with existing cell-cell attachments; another defines the attachment probability of single cells that have no cell-cell attachments. Their values range from 0 (never) to 1 (always). Open circles: in vitro measures; filled circles: attachment probabilities of 0.0/0.0 (single/clustered); triangles: 0.25/0.25; x: 0.5/0.5; diamonds: 0.75/0.75; squares: 1.0/1.0. Cells failed to form any cluster or ALC when both probabilities were set to 0. Changing the cell-cell attachment probabilities had virtually no effect on ALC growth in cultures of up to ∼3,000 cells (1.5×10^5^ cells/cm^2^ in vitro). However, at higher initial cell densities, final ALC diameter increased monotonically with increasing cell-cell attachment probabilities. Attachment probabilities of 1 led to an almost exponential increases in ALC size. Changing the attachment probability of single cells caused extensive changes in ALC size, compared with when clustered cell attachment probability was changed (inset). ALC diameters represent mean values of 100 Monte Carlo runs each executed for 100 simulation cycles (∼6.1 days in vitro). Within simulation, cells and clusters were directed to migrate randomly at the speed of 1 grid unit/time step.

We expected that the kinetics of AT II cell-cell adhesion in vitro may vary between single isolated cells and cells that already had existing intercellular junctions. Analogues used two parameters to control the probability of attachment: one used by single cells and the other by clustered cells. Upon collision, cells always attached when the parameter values were set to 1. Cell attachments were blocked when the parameter value was 0. We varied both from 0 to 1, and documented changes in aggregation and ALC formation. All other parameters were set to their Table 2 values. Results are shown in Fig. 10 for the random migration mode. The conclusions were the same from experiments using the other two migration modes. During simulations, inhibiting or enhancing cell-cell attachment did not alter the capability of a cluster to develop into an ALC. Executions of axioms governing rearrangements of cells within clusters were not affected.

Alteration of cell-cell attachment probabilities induced the changes in final ALC diameters shown in Fig. 10, but did not improve the simulation outcome sufficiently to achieve SM-6. At initial densities of 600–3,000 cells (3 to 15×10^4^ cells/cm^2^ in vitro), increasing or decreasing attachment probabilities had no significant effect on ALC diameter. However, setting the value to zero blocked ALC formation, because there was no aggregation. When the attachment parameter value was set to 0.25 or less, cells failed to form ALCs at the lowest density, corresponding to 1.0×10^4^ cells/cm^2^ in vitro. The small clusters that did form failed to accrete and so failed to develop into ALCs. Changes were most striking above initial densities of 3,000 cells, corresponding to 15×10^4^ cell/cm^2^ in vitro. Cell attachment parameter values for non-clustered cells were the primary driver at higher initial cell densities (Fig. 10, inset). Increasing the attachment probability of clustered cells had a relatively small effect. For example, cells with single and clustered cell attachment probabilities of 0.75 and 0.5, respectively, developed notably larger ALCs than did those with corresponding cell attachment probabilities of 0.5 and 0.75 (Fig. 10, inset).

The observed results were somewhat unexpected but could be explained by analyzing analogue execution. For sparse cultures, ALC diameters were not sensitive to changes in cell aggregation. For a cluster to develop into an ALC, it must have a minimum of six member cells. Most aggregates that formed in sparse cultures contained fewer cells; they remained as clusters and were not included in the ALC measurements. When we increased cell-cell attachment probabilities, the number of clusters and their average size increased but not enough to affect the final ALC diameter. In densely populated cultures, the changes in cluster size were large enough to alter final ALC diameters. However, increased adhesion probabilities often led to formation of a few large unorganized clusters that expanded into oversized ALCs. In some cases, when the cell-cell attachment probabilities were set to 1, all cells aggregated into a single cluster which grew to an ALC with a diameter of >200 µm. Such huge ALCs were not seen in vitro. Such occurrences explain the exponential rise in the measured ALC diameter following an increase in the initial cell population.

### Decreases but not increases in cluster migration speeds had dramatic consequences

**Figure 11:**
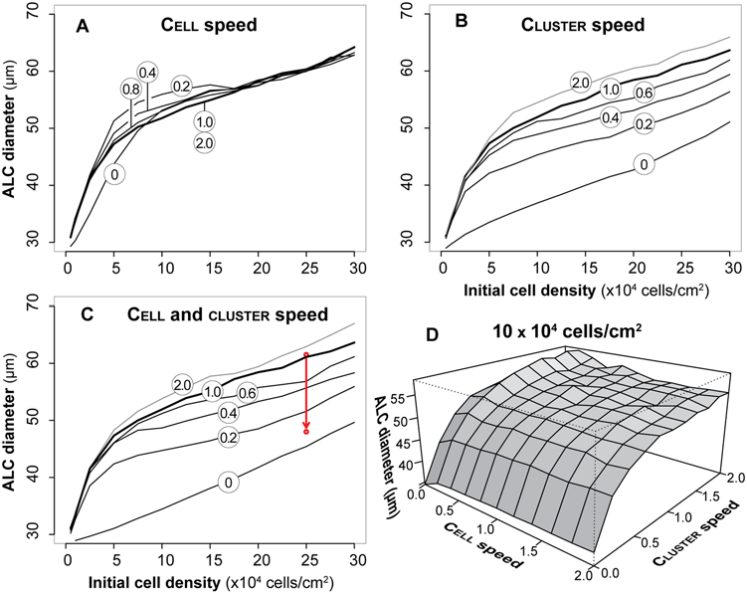
Altered mean ALC diameters following changes in cell and cluster migration speeds. All analogues used the cell density-based migration mode and were parameterized as in Fig. 6B. Values are mean diameters from 100 Monte Carlo runs, each executed for 100 simulation cycles. Single and collective cell migration speeds were controlled parametrically. The speed in Table 2 is 1 grid unit/simulation cycle (which maps to ∼8.5 µm/1.5 h), corresponding to the AT II cell speed in 2% Matrigel. Cluster migration implements collective cell migration. (A) Shown are the consequences of altered cell speed, when cluster speed was fixed at 1.0. (B) Shown are the consequences of altered cluster speed, when cell speed was fixed at 1.0. (C) Shown are the consequences of having cluster and cell speeds be identical, and changing both. The arrow pointing down shows the observed change in mean ALC diameter at the indicated initial cell density when Matrigel density was increased from 2% to 10%. (D) Shown are the consequences of altering cell+cluster speed at the indicated initial cell density. Although the diameter ranges were different, the general pattern was similar at the four other cell densities studied in vitro.

AT II cell and cluster migration speed was an important determinant of aggregation and ALC formation in 3D cultures. Intuitively, one would expect to form larger ALCs by elevating cell and cluster migration speeds. Doing so would increase the cell collision rate and thus accelerate aggregation. Conducting such experiments in vitro is infeasible because a minimum level of Matrigel is required to sustain normal AT II cell behaviors. Testing that hypothesis for AT II analogues was straightforward. We varied cell and cluster migration speeds in a series of simulation experiments. Parameter values specified the mean migration speed of cells or clusters in grid units per simulation cycle. We varied that value from 0 to 2 in increments of 0.2 grid units per simulation cycle. Migration was abolished when the parameter value was set to 0. A speed of one grid unit per cycle, which was used to obtain the results in Fig. 6, maps to the observed, average speed of AT II cells in 2% Matrigel; we refer to that speed as normal in the paragraph that follows. We explored the consequences of altered migration speeds using each of the three migration modes alone. Other parameter values were set to the values listed in Table 2. The results are graphed in Fig. 11 for the cell density-based mode of movement, and in Figs. S1 and S2 for the other two modes.

Using different cell and/or cluster speeds with the cell density-based movement mode elicited noticeable changes in ALC development (Fig. 11). Blockage of single cell migration caused only a small reduction in final ALC diameters in sparse cultures of up to ∼1,500 cells (7.5×10^4^ cells/cm^2^ in vitro), but had no effect in denser cultures (Fig. 11A). When speed was reduced from the normal value of 1 grid unit per simulation cycle, we observed an unexpected, small increase in the final ALC diameters for cultures having initial densities up to ∼3,400 cells (17×10^4^ cells/cm^2^ in vitro), which could be attributed to the slow movement of isolated cells, which allowed enough time for nearby clusters to close in and cohere with the cells. Reduced cell speed had no observable effect in cultures that had initial densities >3,400 cells. We also observed no significant changes in the mean ALC diameters when we doubled cell migration speed: the mean ALC diameter at any cell density remained virtually unchanged.

Slowing cluster migration, while keeping cell migration speed at the normal value, led to a monotonic decrease in the final ALC sizes at each initial cell density (Fig. 11B). Blocking cluster migration did not prevent ALC formation because single cells eventually attached to clusters. Doubling cluster migration speed (from 1 to 2 grid units per simulation cycle) caused only small changes in stable ALC diameters.

Slowing cell and cluster migration speeds together was expected to reflect changes in Matrigel densities. The results (Fig. 11C) were essentially identical to those when cluster migration speed was changed while keeping cell migration speed normal (Fig. 11B). These results predict a dramatic reduction in ALC formation when the ECM is stiffened. The prediction was tested in vitro at a high, initial cell density (Fig. 11C). When the ECM density was increased, cells and clusters aggregated less and formed smaller ALCs [6].

Analyzing analogue execution revealed that in dense cultures, most cells were initialized in close proximity to one another so they were able to form clusters and condense rapidly. The resulting, initial clusters were usually large enough to develop into ALCs without further aggregation. Consequently, cell migration played a minor role. In sparse to moderately populated cultures, cells and clusters migrated over longer distances to aggregate so migration played a more significant role. However, faster movement along with more frequent collisions was offset largely by a smaller time window for forming cell-cell attachments before moving apart. Overall, the results suggested that the cell migration speed in Table 2 might be close to optimal.

Identical simulation experiments using analogues that relied on the chemotactic mode only, gave similar results (Fig. S1), but there was a noteworthy difference. Changing cell migration speeds above zero, while keeping cluster migration speed fixed at the normal value, failed to alter ALC diameters at any of the initial densities tested. Changing cell and/or cluster migration speeds using cells that relied on the random migration mode only (Fig. S2) also failed to significantly alter final ALC diameters: changes, relative to those in Fig. 11, were muted.

## Discussion

Epithelial morphogenesis is tightly orchestrated in time and space. Understanding its complexities remains a challenge. Study of relatively simplistic, yet increasingly realistic, in vitro cell culture systems continues to provide mechanistic insight at multiple levels. Primary human AT II cells cultured in 3D Matrigel is a recently developed system. Its phenotype recapitulates several basic features of mammalian alveolar morphogenesis and regeneration [6]. Each cell's molecular biology appears manifest in a relatively small set of environment-dependent, epigenetic principles. What are those operating principles? How do they come about? Our knowledge of the organization of mechanistic details and how they emerge at the cell level is still too sparse to begin answering those questions directly.

To help gain a better understanding, the approach developed and applied herein has been to build concrete analogues that exhibit key phenotypic attributes in common with those of AT II cell cultures. In mature form, the analogues' mechanisms and operating principles can stand as plausible representations of—working, dynamic, explorable metaphors for—those of AT II cells. To that end, we have created, studied, and improved quasi-autonomous AT II cell-mimetic analogues that exhibit the identified attributes in Table 1, while adhering tightly to the small set of axiomatic operating principles illustrated in Figs. 3 and 4.

Achieving the targeted attributes along with envisioned, future uses and capabilities dictated model design and implementation. We approached the problem from a cell-level perspective by viewing multicellular structure development in terms of epigenetic events, cellular activities, and their interactions, with an understanding that molecular and biophysical details, as well as other sub-cellular information, conflate into the cell-level mechanisms and events. So doing allowed us to focus on aspects that directly map to available biological information and obviated the need to reduce the cellular phenomena into more detailed molecular or physicochemical representations, such as those developed in [16], [17], and [18]. For the attributes targeted, high-resolution chemical or physical details were not needed. However, cell and system design features make it easy to add detail when it becomes necessary to do so.

From an engineering perspective, AT II cells are considered independent entities that act autonomously driven by their own internal mechanisms interacting with the surrounding environment. Each cell decides what to do, and when, using information gathered at its interface with the environment. There is no global mechanism or controller directing cell actions; nevertheless, they are able to self-organize reliably into coherent multicellular structures. To better approximate that ability, we required that cells have internal, local control over their own actions. That meant, to the extent possible, no global control or intervention dictated cell action. We also required that cells be responsible for scheduling and executing their own action events. Those requirements precluded model construction based exclusively on established, formal cellular automata [19], [20], [21], cellular Potts models [22], [23], [24], or agent-based methods [25], [26]. While the AT II cell cultures are grounded in agent-based modeling methods, and share similarities with cellular automata and cellular Potts models, they cannot be described strictly as being any of the three model types. Because all are ultimately based on object-oriented programming methods, AT II cell cultures can be considered generalized constructions in the object-oriented domain.

Simulation outcomes in Fig. 5 demonstrated that cells self-organize and develop structures that closely resemble referent ALCs. Their growth phenotype mirrored dynamic developmental patterns of AT II cells in vitro. Even though abstract, the analogue's phenotype supports the idea that in vitro cytogenesis may be explained by a small number of generative principles adhered to tightly by each individual cell. Also notable are the critical roles of a small number of cell axioms, which were not apparent from their usage frequencies. Their importance in proper ALC development became obvious when their use was blocked during simulation. As one might expect, functional blockage of a more frequently used axiom like 2b prevented aggregated cells from forming ALCs. What we failed to anticipate was the marked disruption of proper ALC morphogenesis when execution was blocked of an infrequently used axiom like 2f or 3a. The consequences demonstrated how a relatively small impairment in the causal principles of operation could produce an abnormal global effect. To the extent that the in silico to in vitro mappings shown in Fig. 1 are valid, we speculate that the analogue's phenotype may have an in vitro counterpart. On the other hand, the phenotype becomes automatically invalid when extrapolated to AT II cells in vivo, whose characteristics and properties such as robustness are beyond the scope and capabilities of the AT II analogues developed herein.

Of the three migration mechanisms tested, cell migration based on local cell densities yielded data that were most similar to the referent data (Fig. 6). The encoded mechanism allowed cells to maintain persistent directionality in a densely populated culture while exhibiting the collectively convergent patterns observed in AT II cultures. The method was not as effective when cells were sparsely populated, and required some random movement to achieve SM-6. Compared to the cell density-based migration, chemotaxis was a somewhat less effective driver of aggregation, especially in dense cultures in which cells lost directional persistence due to rapid fluctuations in local attractant levels. The observed differences could be attributed to the analogue's chosen spatial discretization (e.g., hexagonal vs square) and implementation details, or unknown artifacts. In vitro and in vivo chemotaxis also could involve local gradients of multiples chemotactic agents, which additionally might modulate expression of cell surface molecules involved in cell aggregation. Consequently, we do not preclude the possibility that enabling a correspondingly more detailed chemotaxis mechanism might further improve cell aggregation characteristics, and nullify the observed differences in outcome.

Nevertheless, the overall results suggest the possibility that a mechanism other than chemotaxis might be an important driver of AT II cell aggregation in 3D matrix. The cell density-based migration mechanism does not yet map to a specific, known mechanism. It is an abstract placeholder for whatever non-chemotactic mechanisms enable AT II cells to sense other cells in their surroundings, obtain directional cues, and migrate based on that information. One mechanism could involve matrix remodeling and sensing by the AT II cells similar to how collagen remodeling is thought to guide the invasive migration of neoplastic mammary epithelial cells [27], [28]. Another could involve direct or indirect long-range intercellular connections. Mouse limb bud and *Drosophila* wing imaginal disc cells in vitro can rapidly develop actin-based intercellular extensions as long as 700 µm [29]. Similar long-range intercellular structures have been documented in cell cultures of different lineages [30]. Cellular extensions physically connecting cells over shorter distances have been observed during morphogenic development of several systems [31]. Such structures could provide directional cues for AT II cell migration. Interestingly, Videos S4-6 show instances of filamentous extensions that appear between cells or clusters of cells during aggregation. It is also possible that AT II cells in vitro dynamically employ multiple motility mechanisms, a hypothesis supported by the simulation results (Fig. 6). In AT II culture videos, some cells appeared to switch between random and directional movements, while others exhibited an apparently single mode of migration; for example, see Video S6 beginning ∼10 h through the recording. In other epithelial cell lines, including MCF10A, counter modes of migration—random versus directionally persistent—are regulated by an internal mechanism dependent on Rac1 protein activity [32]. As specific information becomes available, it can be added to the set of targeted attributes to stimulate additional rounds of mechanism refinements.

The above discussion highlights an important feature of this model class. Additional attributes and details can be added during the iterative model refinement process to reflect new biological information or in vitro context. So doing will help strengthen the in silico to in vitro mappings at all three levels illustrated in Fig. 1 while increasing the predictive capacity of the AT II analogues. On the other hand, establishing similar in silico to in vivo mappings is expected to be challenging. Lacking reliable mappings, the analogue's phenotype described herein cannot be extrapolated to an in vivo context. With sufficiently mature analogues, we envision the same iterative refinement process being undertaken to evolve analogues to simulate and predict AT II cell behaviors under physiological and pathological conditions.

Finally, axiom use results in Fig. 9 show that at the same time, different cells within the same culture are engaged in quite different activities. Assume that the same is true in vitro; for example, one AT II cell can be moving about within a cell cluster while another is migrating alone, and another is trying to attach itself to an early state ALC, etc. It seems reasonable that the ensemble of molecular biology details, such as gene and protein expression levels, which enable those different activities could themselves be different. For example, single cells actively migrating might be upregulating expression of genes and proteins optimized for cell locomotion, while those establishing cell-cell attachment are upregulating expression of adhesion molecules such as E-cadherin necessary for intercellular junction organization. At the same time, cells composing a mature ALC exhibit a stable polarized phenotype, and resume surfactant and lamellar body secretion which could lead to proteomic profiles that are different from those of single migrating cells. Patterns detected in such data averaged over all cells may have little scientific value in answering such questions as these. When and how does an AT cell choose to switch from one activity to another? Why does it choose one action rather than another? Are several action options always available to each cell? Obtaining plausible answers to these questions is essential to achieving new and deeper insight. The class of models presented herein provides a rigorous platform to hypothesize, challenge, and refine plausible answers. The causal chain of events responsible for most simulation events can be explored in detail, and assessments can be made as to whether critical events are biotic (supportable by in vitro evidence) or not.

In summary, the main significance of this study is the finding that AT II cyst formation can be accomplished by cells' tight adherence to a small number of axiomatic operating principles, which may map to biological counterparts underpinning in vitro cystogenesis. We project future rounds of model refinement and validation, for additional attributes or use, will help further strengthen the mappings in Fig. 1 and provide a viable, productive strategy to unravel mechanistic bases of epithelial morphogenesis.

## Supporting Information

Text S1 Supplemental Methods.
(0.18 MB PDF)

Figure S1 Altered alveolar-like cyst (ALC) growth in silico following changes in simulated cell and cluster speed (chemotactic mode). Single and collective cell migration speeds are controlled parametrically. Cluster migration implements collective cell migration. Individual cells and clusters were directed to migrate chemotactically along a local cell-produced attractant gradient. All other model parameters were set to the Table 2 values. Increasing or decreasing cell speed had a material effect on ALC growth. (A) Reduced single cell speed; (B) increased single cell speed; (C) reduced cluster speed; (D) increased cluster speed; (E–F) simultaneous reduction/increase in single/collective cell speed. We executed 100 Monte Carlo runs per cell density; each run lasted 100 simulation cycles.
(8.80 MB TIF)

Figure S2 Altered ALC growth in silico following changes in simulated cell and cluster speed (random migration mode). The experiments are the same as those in Fig. S1 except for the migration mode used.
(8.82 MB TIF)

Video S1 Simulated ALC morphogenesis with an initial population of 500 simulated cells (∼2.5×10^4^ cells/cm^2^ in vitro). Model parameters were set to the Table 2 values. Seeded randomly across the culture grid space, the cells migrated randomly and aggregated into coherent clusters. Sufficiently large clusters grew luminal space and developed into ALCs with the luminal space enclosed by a monolayer of cells. We used uniform hexagonal grids to represent culture space and composing elements, so a hexagonally shaped ALC in silico approximates a roundish cyst in vitro. Regular hexagons with white center depict individual cells. Gray and black spaces represent matrix and free (or luminal) space respectively.
(0.88 MB MOV)

Video S2 Simulated ALC morphogenesis with an initial population of 1,000 simulated cells (∼5×10^4^ cells/cm^2^ in vitro). The experiment is the same as that in Video S1 except for the initial cell population size.
(1.26 MB MOV)

Video S3 Simulated ALC morphogenesis with an initial population of 5,000 simulated cells (∼25×10^4^ cells/cm^2^ in vitro). The experiment is the same as that in Video S1 except for the initial cell population size.
(1.82 MB MOV)

Video S4 ALC morphogenesis in vitro with an initial cell density of 2.5×10^4^ cells/cm^2^. AT II cells were grown in Matrigel culture for six days. Images were collected at 15 min intervals beginning 8 h after plating.
(4.89 MB MOV)

Video S5 ALC morphogenesis in vitro with an initial cell density of 5×10^4^ cells/cm^2^. The experiment is the same as that in Video S4 except for the initial cell density.
(7.52 MB MOV)

Video S6 ALC morphogenesis in vitro with an initial cell density of 10×10^4^ cells/cm^2^. The experiment is the same as that in Video S4 except for the initial cell density.
(6.38 MB MOV)

Video S7 Simulated ALC formation. Cells migrated chemotactically along a cell-produced attractant gradient. The initial cell population was set to 1,000 cells (∼5×10^4^ cells/cm^2^ in vitro). The experiment is the same as that in Video S1 except for the initial cell population size and the migration mode used.
(0.88 MB MOV)

Video S8 Simulated ALC formation. Cells migrated along a local cell density gradient. The initial cell population was set to 1,000 cells (∼5×10^4^ cells/cm^2^ in vitro). The experiment is the same as that in Video S1 except for the initial cell population size and the migration mode used.
(0.78 MB MOV)
