# Supplementary material for: A Computational Approach to Understand In Vitro Alveolar Morphogenesis
Source: PLoS One. 2009 Mar 13;4(3):e4819. doi: 10.1371/journal.pone.0004819 (PMC2653231; doi:10.1371/journal.pone.0004819)
Supplement: Text S1 — Supplemental Methods. (0.18 MB PDF) [file pone.0004819.s001.pdf]

Supplementary Material
for
A Computational Approach to Understand In Vitro Alveolar Morphogenesis

Sean H. J. Kim, Wei Yu, Keith Mostov, Michael A. Matthay, and C. Anthony Hunt

Methods

Model design requirements

To achieve both short and long term goals, we needed models and components that met the following five basic requirements. 1) Model components must be modular. Components, analogous to referent counterparts, are designed to interact. It must be easy to join, disconnect, or replace components without altering the functions and interrelations of other components. 2) The composed system must be flexible. It must be relatively simple to change usage and assumptions, or increase or decrease analogue detail in order to meet the particular needs of a new experiment, without requiring significant system reengineering. 3) System components must be reusable and adaptable for simulating epithelial cell behaviors in different experimental conditions in the presence and absence of treatments and interventions. 4) Analogues need to be articulated, multi-component systems that can be expanded easily to represent additional details or mimic additional referent attributes. 5) Components and methods need to be designed so that they can be replaced easily with alternate or more detailed counterparts, when needed.

Agent-based and discrete event simulation methods

To achieve the above requirements, we adapted agent-based modeling (ABM) [1], discrete event simulation (DES) [2,3], and aspect-oriented software development methods [4]. In ABM, a system is comprised of quasi-autonomous, decision-making entities called agents. Each agent follows a set of rules that governs its actions and interactions with other system components. ABM facilitates creating systemic behaviors and attributes that arise from the purposeful interactions of components. Agent-based models have advantages when attempting to understand and simulate phenomena produced by systems of interacting components. We used quasi-autonomous agents to represent epithelial cells. We also employed quasi-autonomous components outside of the simulated biological system, but still within the computational framework. They played procedural, observational, and data analysis roles analogous to those preformed by researchers conducting experiments. In DES, system operation is represented as a temporal sequence of events, each occurring at an instant of time. System state evolves during discrete time intervals. Using DES methods facilitated encapsulating system operations and conceptualizing complex dynamics. The methods provided a rigorous formalism for managing modularity and hierarchy. From a simulation perspective, cellular activities such as cell division, death, and adhesion could be represented as being discrete. Operations of all analogue components were represented as discrete events. Stochasticity, which is natural to both ABM and DES, was introduced mostly in the form of probabilistic parameters that regulated agent actions and execution order.

CLUSTER component

A CLUSTER is an agent and composite object. It is an aggregate of CELLS and has its own actions. A CLUSTER is created when two or more CELLS attach. Single CELLS that establish attachments to member CELLS are added to the CLUSTER. The CLUSTER is deactivated and withdrawn when membership diminishes to one; the remaining CELL reverts to single CELL status. Each CLUSTER uses an identical step function to determine its action. The step function is scheduled every simulation cycle. A CLUSTER can

either migrate a certain distance or do nothing. Migration speed and the probability of actual movement are specified parametrically. Similar to a CELL, a CLUSTER is capable of three types of migration: random movement, CHEMOTAXIS, and CELL density-based migration. A CLUSTER does not have its own means to assess the local environment for directional movement. It simply collects that information from its member CELLS to determine a direction to move: for simplicity, it adopts the majority migration mode of its member CELLS. To simplify design, CLUSTER shape remains static during movement: every member CELL, as well as LUMINAL SPACE, moves the same distance in the direction of migration; CELL-CELL attachments are maintained. CLUSTER movement stops when the movement is blocked by non-member CELLS or other CLUSTERS. The process is an abstract representation of in vitro cluster movement, which can involve significant changes in the overall aggregate shape. However, there is evidence for collective cell migration in which cell-cell junctions and relative positioning of member cells within the group are retained [5]. Representing CLUSTER migration as a single action maps to experimental evidence that cluster motion is not a simple summation of individual cell motion but a collective process of an integrated unit [6]. For general object interactions, each CLUSTER is provided with standard class methods to query and access member CELLS and associated LUMINAL SPACE. Because CLUSTERS do not exist on a separate grid, they do not have or need their own positioning information.

AT II CULTURE

Conceptual abstraction of an in vitro culture as a whole is encoded as CULTURE; it provides space for and access to the abovementioned components. It subclasses from SimState, which represents the simulation proper. It has an event schedule and a generator of pseudo-random numbers (PRNs). A CULTURE inherits the basic simulation components as well as base methods that are called automatically at the simulation's start and end, and it has its own start and end functions that can overwrite the base methods. CULTURE creation begins with a scan of a parameter file that contains parameter specifications defined automatically by EXPERIMENT MANAGER or manually. CULTURE dimensions and, when applicable, the size of initial CELL population are specified in its parameter file. That file also contains specifications for CELL migration. Following parameterization, the start function is called immediately prior to the start of simulation to initialize a CULTURE and create its components according to specifications. The CULTURE uses standard two-dimensional (2D) hexagonal grids to provide the space in which its objects reside. An additional grid can be added to simulate diffusion when needed. Grids have toroidal topologies. Representation of three-dimensional (3D) space can be achieved when needed using stacked 2D grids, or alternatively with a 3D cubical or octahedral grid, although the computational cost of the latter is steep. Once the grid is initialized, CULTURE components—CELLS, MATRIX, and FREE SPACE—are placed on the grid and an initial scheduling of CELLS is created on the master event schedule. For simplicity, each grid position is occupied by one object. That condition can be easily changed when the need arises. Simulation starts following completion of the CULTURE start function. As execution advances, the event schedule is stepped for a number of simulation cycles or until a stop signal is produced. Simulation time is advanced discretely, and is maintained by the schedule. Ordering of events to occur within the same simulation cycle is pseudo-random. Within a simulation cycle, every CELL is given an opportunity to interact with adjacent objects in its environment. At the end of simulation, the CULTURE finish function is executed to close remaining open files and clear the system.

The high level EXPERIMENT MANAGER

EXPERIMENT MANAGER, the top-level system component, is an agent that provides experiment protocol functions and specifications. The specifications define the mode of experimentation and the system's parameter vector. Experiments can be conducted in default, visual, or batch mode. An experiment in default mode is simply a single execution. In visual mode, a CULTURE GUI is created and the visualization console launched. Batch mode enables automatic construction and execution of multiple experiments, as well as processing and analysis of recorded measurements. EXPERIMENT MANAGER has user-defined specifications to delimit the parameter space from which individual CULTURE parameter files

are generated. For example, the upper and lower bounds and in-between increments of CELL-CELL attachment probabilities described below, or CELL migration speeds can be specified. Individual parameter files created by EXPERIMENT MANAGER can have different parameter values for CELL-CELL attachment, CELL migration speed, etc. A CULTURE's grid dimensions, initial CELL population, and CELL migration mode can also be varied similarly between parameter files. Once parameter files are generated, EXPERIMENT MANAGER automatically executes a batch of experiments, each corresponding to a different parameter file. The user specifies the number of repetitions for each experiment. Repetitions are executed sequentially. Parallel execution of individual repetitions requires additional system protocols. After completion of all experiments, basic analytic operations are used to collect and summarize experimental data.

AT II CELL design

AT II CELLS mimic specified behaviors of alveolar type II cells in cultures. They are quasi-autonomous agents that follow their own agenda and schedule their own events. They have decision logic and axiomatic operating principles (Figs. 3 and 4) for interacting with the neighboring environment. They have a step function, which assesses all neighboring objects and executes an appropriate action. To achieve the initial set of target attributes, we defined what we judged to be a minimal set of actions: attach to an adjacent CELL, migrate, and rearrange within a CLUSTER. Every CELL maintains a state variable indicating whether it is a member of a CLUSTER. When stepped, a non-clustered CELL can either migrate or attach to an adjacent CELL. It migrates if it has no adjacent CELLS. When stepped, a clustered CELL can form an additional CELL-CELL attachment or rearrange its position within its parent CLUSTER. The rearrangement action is available only to clustered CELLS. CELL-CELL attachment action is available in either state, but the probability of attachment to a neighboring CELL can vary parametrically between non-clustered and clustered CELLS. The parent CLUSTER dictates collective migration of the clustered CELLS.

CELL migration

CELLS have available three migration modes: random movement, CHEMOTAXIS, and CELL density-based migration. Epithelial cells in vitro can adopt different modes of migration in response to changes in cellular state or environment [7]. Random motility is characterized by the absence of persistent directionality. Random motility, apparent or true, may involve different molecular and cellular mechanisms, although much remains unknown. For simplicity, random AT II CELL movement is implemented as a random walk. When stepped, a CELL in random migration mode selects randomly one of six target directions and moves only if the target grid location is CELL-free. AT II cells, like other epithelial cell types, exhibit directional migration patterns in vitro. Different mechanisms and stimulus types are thought to contribute to guided cellular movement. Examples of taxis include chemotaxis, haptotaxis (adhesion gradients), and thigmotaxis or stereotaxis (physical contact). AT II cells in vitro can adopt the chemotactic mode in the presence of a chemoattractant [8]. AT II CELLS encode a migration mechanism that mimics chemotaxis. When in CHEMOTACTIC mode, a CELL, when given its opportunity to update each simulation cycle, completes two tasks. First, it produces an amount of ATTRACTANT and adds it to the corresponding grid location in the DIFFUSER grid space. The amount is a random value between the minimum and maximum levels specified in Table 2. The CELL then evaluates ATTRACTANT levels in the six adjacent DIFFUSER grid locations and identifies the space having the highest level. When more than one adjacent site has the same highest level, one is selected randomly. The CELL moves to the corresponding location in CULTURE space only if that site is CELL-free. CELLS can also be specified to migrate away from the ATTRACTANT.

Observations of Madin-Darby canine kidney (MDCK) cells demonstrate that mechanisms other than chemotaxis can drive directional migration [7]. In the studies of AT II cells in 3D cultures [9] and wounding assays [10], single cells and cell aggregates exhibit directionally persistent migration patterns but the underlying mechanisms are unknown. Multiple mechanisms may be involved. One can speculate, for example, that cell detection of directional changes in adjacent matrix viscoelastic properties [11] and

other known mechanisms [12] may play a role in directional AT II cell movement in 3D matrix. We implemented CELL density-based migration to explore plausible but not yet verified mechanisms. The algorithm closely resembles simulated chemotaxis. When stepped, the migrating CELL computes the CELL population within each of six, identical-sized, adjacent neighboring regions; each region corresponds to a direction in the hexagonal grid. The CELL moves toward the most densely (or sparsely, if so specified) populated region. The move is executed only if the adjacent grid location in the selected direction is CELL-free.

Every CELL maintains a state variable specifying its current migration mode. Migration modes are encoded as separate object methods. CELLS can switch their migration mode during simulations, for example from random movement to CHEMOTAXIS when the ATTRACTANT is detected. If migrating, the CELL executes a move method corresponding to the current migration state. CELLS also have a parameter that can be used to introduce random movements while CELLS are in a directional migration mode. The parameter specifies the probability of electing to move randomly when the CELL is in directional migration mode. The default value of zero prevents any random movement when the CELL is in directional mode. The parameter is not used by CELLS in random mode. CELL migration speed is specified parametrically. The parameter specifies the *average* CELL speed in grid units per simulation cycle. A parameter value = 1 results in an average speed of one grid unit per simulation cycle. Setting the parameter to zero abolishes migration. Non-integer parameter values (e.g., 0.5 or 2.8) require approximation. In current implementation, non-integer speeds are resolved as follows. A CELL specified to migrate 0.5 units per cycle has a 50 percent chance of moving one unit at each cycle. Consequently, its instantaneous speed is either zero or one unit per cycle. However, on average, the CELL moves 0.5 units per cycle. Following the same logic, a CELL specified to migrate 2.8 units per cycle moves two units and then has an 80 percent chance of moving another unit. The same method is used to accommodate non-integer CLUSTER migration speeds.

CELL-CELL attachment

CELL-CELL attachment can occur when CELLS contact. The probability of attachment is varied parametrically. One parameter controls the probability of CELL-CELL attachment initiated by a non-clustered CELL; a second specifies the probability of attachment by a CELL in a CLUSTER. When selected to update its status, a CELL first determines if it has unattached, adjacent CELLS, and if it does, draws a PRN from a uniform distribution, $U(0,1)$. Attachment to a randomly selected CELL neighbor occurs only when the PRN is less than the probability threshold set by the parameter corresponding to the current CELL state. Newly attached CELLS are then updated to clustered state. If one is a non-clustered CELL while the other is a member of a CLUSTER, the non-clustered CELL is simply added to the existing CLUSTER. Two CLUSTERS merge if a member CELL attaches to a CELL in another CLUSTER. When CLUSTERS merge, the CLUSTER of the initiating CELL remains active while the other CLUSTER is deactivated and removed from the schedule; all members of the deactivated CLUSTER are added to the remaining CLUSTER.

Clustered CELL rearrangement

CLUSTER members change their positions to achieve a more favorable neighboring object configuration. CELL rearrangement activities are specified using axioms. An axiom specifies a precondition and corresponding action. Preconditions are defined in terms of neighboring object configurations. The method for preconditions specification evolved as follows. We first enumerated all possible compositional combinations of neighboring objects. We did so by constructing a truth table of three logical variables (true if present) corresponding to the three types of CULTURE components—CELL, MATRIX, and FREE SPACE. For each distinct composition, we subdivided its possible configurations into non-overlapping groups using simple classifiers, each group corresponding to a precondition. Having all neighbors of one component type is a single configuration, which was specified as a precondition (1a-c, Fig. 4). A neighboring composition of two or three component types has multiple possible

configurations. For the composition consisting of only CELLS and MATRIX, we used the number of neighbor CELLS and their adjacency as classifiers. For example, configurations containing three CELLS and three MATRIX objects were grouped and specified as a precondition. Another is a precondition of two nonadjacent CELLS and four MATRIX objects. A neighborhood composition of CELLS and FREE SPACE, or MATRIX and FREE SPACE, was specified as a precondition without further classification. Configurations containing all three component types defined three preconditions (3a-c, Fig. 4). One corresponds to configurations having one MATRIX object. The second comprises configurations having one CELL in contact with a MATRIX object. The last combines the remaining configurations consisting of all three-component types. While the preconditions are specific to hexagonal grid implementation, the protocol provides a general encodable approach to cataloguing possible neighboring object configurations and defining preconditions for other types of 2D grids, as well as for 3D grid representations or grid-free networks. However, 3D grid representation most likely will require a computational algorithmic approach due to an exponential increase in the number of possible configurations.

Every precondition was assigned one of two operations: relocate or remain in place. We used observations reported in the literature to help avoid axioms that may have been judged abiotic and to prefer axioms for which supporting evidence was available. Absent evidence, variations of an axiom were implemented and the consequences (in silico predictions) observed upon execution. Ones that moved the analogue closer to validation were selected for the next round of refinement. That process of iterative instantiation, rejection (or acceptance), and revision of axioms along with concurrent revision of the preconditions yielded the following axioms (illustrated in Fig. 4), whose preconditions refer to the composition of six neighboring objects.

1a. CELLS only: push an adjacent CELL and move to its location; leave behind a FREE SPACE. If the pushed CELL has a neighbor inline with the direction it is being pushed, then the neighbor too is pushed. A straight-line chain of *n* CELLS can be pushed; we arbitrarily set $n = 5$. If $n > 5$, then the initiated action fails, and that CELL waits until its next update opportunity in the next cycle. When $n \leq 5$, the MATRIX or FREE SPACE at the end of the chain of CELLS is removed, and the leading, pushed CELL occupies that location.
1b. MATRIX only: do nothing
1c. FREE SPACE only: exchange places with an adjacent FREE SPACE
2a. MATRIX plus one CELL or two adjacent CELLS: exchange places with an adjacent MATRIX that is next to a CELL
2b. MATRIX and two nonadjacent CELLS: remove an adjacent MATRIX that is next to a CELL neighbor; move to that location while dragging the other attached CELL; leave a MATRIX in the attached CELL'S location. If the CELL being dragged has a neighbor inline with the direction it is being pulled, then subsequent events are as in 1a.
2c. MATRIX and three CELLS: do nothing
2d. MATRIX and four CELLS: exchange places with either adjacent MATRIX
2e. MATRIX and five CELLS: remove the MATRIX; move to its location; leave behind FREE SPACE
2f. CELLS and FREE SPACE only: push an adjacent CELL and move to its location; leave behind a FREE SPACE. If the pushed CELL has a neighbor inline with the direction it is being pushed, then subsequent events are as in 1a.
2g. MATRIX and FREE SPACE only: do nothing
3a. CELLS, FREE SPACE, and a single MATRIX: remove the MATRIX; move to its location; leave behind a FREE SPACE
3b. FREE SPACE, MATRIX and a single CELL located next to a MATRIX neighbor: remove a FREE SPACE; while dragging an attached CELL, move to that location. If the CELL being dragged has a neighbor inline with the direction it is being pulled, then subsequent events are as in 1a.
3c. All other configurations containing CELLS, MATRIX and FREE SPACE: do nothing

Single object neighborhoods

Axioms 1a-c correspond to single object neighborhoods. A CELL inside a CLUSTER may find itself surrounded by other CELLS. Experimental evidence suggests that AT II cells in such a predicament produce lumen by exocytosis and strive for cell separation [9]. Based on that evidence, we specified Axiom 1a: it dictates that the surrounded CELL separate away from adjacent CELLS and create a LUMINAL SPACE in its place. The process involves pushing against and moving a randomly selected neighbor and then relocating to its position. Axiom 1b specifies doing nothing when the rearranging CELL is surrounded only by MATRIX. Some CELLS in a developing alveolar-like CYST (ALC) can be isolated within the LUMINAL SPACE, corresponding to the precondition of Axiom 1c. While MDCK and MCF-10A cells inside cyst lumen have been shown to be apoptotic [13], we know that AT II cells do not undergo significant apoptosis [9]. Absent any additional evidence, we imposed a simple random walk reflecting that such a CELL will strive to improve its local environment.

Neighborhoods containing two object types

Axioms 2a-g stipulate CELL actions when there are just two neighbor types. Preconditions of Axioms 2a-e represent neighborhoods of CELLS and MATRIX but no FREE SPACE (see Fig. 4). From observation of an early analogue during evaluations, we learned that cells have such neighboring configurations during cluster or cord formation. We initially classified cell and matrix arrangements based on the number of neighboring cells, and used that information to define a corresponding axiomatic precondition. So doing produced five preconditions each corresponding to one-to-five neighboring CELLS. Of course, if we discretize space differently, the classification would be different, as would the axioms. However, the key behaviors and end points would be expected to be essentially the same. To assign CELL actions, we again studied the time-lapse videos focusing on aspects of AT II cell aggregation and noted that many clumped cells shift their positions within the cluster in an apparently random manner. We specified axioms accordingly so when any of the 2a-e preconditions applied, the CELL needing to make a decision repositioned itself to a randomly selected MATRIX location. However, that simple action often caused the CLUSTER to decompose into clumps. We observed that CLUSTER fragmentation occurred most often in configurations involving one or two neighboring CELLS, and when the decision-making CELL elected to move to a neighboring MATRIX position not flanked by a CELL. Guided by those observations, we made corrective changes to the axioms to prevent the unintended CLUSTER breakage.

When the decision-making CELL has one CELL neighbor, the revised axiom directs that it move to an adjacent MATRIX position flanked by its CELL neighbor. A similar action applies when the CELL has two adjacent CELL neighbors. Both instances are preconditions of Axiom 2a. When the two CELL neighbors are not adjacent, Axiom 2b dictates that the decision-making CELL avoid separating itself from its two neighbors by pulling one along with it as its relocates. If the rearranging CELL has three CELL neighbors, it does nothing as specified by Axiom 2c. Variations of the preceding relocation axioms did not improve simulation outcomes.

Axioms 2d and e have a precondition of four or five CELL neighbors, plus MATRIX. Both axioms specify movement to a randomly selected MATRIX position. Axiom 2e additionally directs that the CELL create a new LUMINAL SPACE as it relocates. Analysis of Axioms 2d and e use during simulations confirmed that neither caused CLUSTER breakage, so we did not explore further revisions. Axiom 2f applies when the decision-making CELL has only CELL and FREE SPACE neighbors. That configuration is often associated with a CELL that is trapped within a LUMEN. To enable such a CELL to improve its condition, we assigned to Axiom 2f the action of moving outward by pushing against a randomly selected CELL neighbor. We tested alternate actions, but all failed to consistently dislodge CELLS from the enclosed LUMINAL SPACE. Axiom 2g specifies the default action of doing nothing when the neighborhood consists only of MATRIX and FREE SPACE; the CELL reverts to a non-clustered state because it has no neighboring CELLS, and subsequently follows single CELL actions. CELLS in developing CYSTS

do not typically find themselves with only MATRIX and LUMINAL SPACE neighbors. Each member CELL maintains at least one CELL-CELL attachment as it moves within the parent CLUSTER.

Neighborhoods containing three object types

Axioms 3a-c specify actions when all three object types are present. One of the targeted attributes is achieving the in silico counterpart of stable, spherical cysts. A sphere in continuous space maps to a regular polyhedron in discretized hexagonal space. The circular cross-section of a sphere maps to the polygonal cross-section of the polyhedron in hexagonal space, use of which is supported by evidence that epithelial cells tend to assume a hexagonal topology [14]. Thus, we reasoned that a hexagonal cyst maps to the roundish shape of a section through a somewhat spherical AT II cyst. A joint requirement was that ALC shapes must be convex. Simulation of early AT II analogues frequently produced non-convex ALC with eccentric shapes similar to the simulated MDCK cysts reported by Grant et al. [15]. Morphological analysis of the in silico shapes indicated that CELLS composing an irregular edge tended to be in an indented position adjacent to all three object types but with only one neighboring MATRIX. Consequently, we specified Axiom 3a to have that precondition and defined the action that the decision-making CELL move outward one space in an effort to increase MATRIX contacts. So doing allowed CELLS to consistently produce ALCS having convex polygonal shapes.

Cyst formation involves lumen enclosure by cohered cells that separate the lumen from extracellular matrix. At least two CELL neighbors are needed to enable the decision-making CELL to partition LUMINAL SPACE from MATRIX in its immediate neighborhood. Such a partition cannot form when the decision-making CELL has only one CELL neighbor. An obvious candidate action to achieve separation of MATRIX from LUMINAL SPACE would be CELL division to create an additional CELL neighbor. However, AT II cells do not undergo detectable proliferation [9]. We speculated that, to form and enclose a lumen, cells simply converge to close gaps. Axiom 3b helps accomplish the analogous result by having the rearranging CELL move to a randomly selected FREE SPACE grid position while dragging along its CELL neighbor. The precondition helps insure that the decision-making CELL is at an open edge. Drawing in the CELL neighbor is implemented as an iterative pulling of the CELL. We found no biological evidence to guide articulation of specific actions for any of the remaining configurations of neighborhood containing three object types. Following the principle of Occam's razor, Axiom 3c thus dictates the default action of do nothing. Some of these configurations satisfy the CELL mandates to achieve three surfaces [13].

Simulating 3D behaviors

Axioms 1a, 2a, b, e, and f are preempted when a decision-making CELL chooses to move off the CULTURE grid. The preemption frequency is specified using a threshold function and parameters. The action is needed to more closely simulate observed 3D phenomena. The recorded images of AT II cell cultures are 2D optical cross-sections. The images do not show cells and other contents that are outside the field of view or out of focus. As larger cysts form, more cells are positioned out of focus; they "disappear" from the focal plane. The CULTURE grid and its contents represent the focal plane of 3D cell cultures. By analogy, some CELLS must be positioned outside the CULTURE grid as cystic structures develop. We devised different strategies to allow CELLS to disappear from the grid but maintain their presence in the simulation. Our objective was to find one that would move the analogue to validation without being abiotic or imposing undue computation or complexity. Our initial strategy was to use a 3D grid to represent CULTURE space and revise the model accordingly. We constructed a basic 3D model in which the CELLS were allowed to aggregate and form CLUSTERS, but not to rearrange within CLUSTERS. Execution and analysis time expanded dramatically, as expected: too much to allow a productive pace of iterative revision and analysis. The approach selected was to use CLUSTERS to keep track of CELLS that belonged to a specific CLUSTER or ALC, but were positioned outside the 2D CULTURE's grid. In the finalized implementation, every CLUSTER maintains a list of member CELLS that lie outside the grid. For simplicity, only clustered CELLS were allowed to relocate off the grid. The decision-making CELL used Axiom 1a, 2a, b, e, or f as exit points. They were specified as exit points to minimize CLUSTER separation

when the decision-making CELL withdrew from the grid. We initially used a ratio-based threshold that imposed a specific ratio of CLUSTER size to its off-the-grid CELL population. CLUSTER size was computed as the number of member CELLS on the grid plus the number of associated LUMINAL SPACE objects. The decision-making CELL computed the ratio of its parent CLUSTER size to the number of off-the-grid member CELLS. If the ratio was greater than the threshold value, the decision-making CELL withdrew from the grid; otherwise, it executed the axiom-specified action. Assuming the clusters had spherical shapes, we specified the threshold to be 0.25, which corresponds to the ratio of bisecting plane area to surface area of a sphere. Use of that threshold in simulations provided some reduction in CLUSTER/CYST size but failed to produce the desired reduction. The method's relative inefficacy was attributed in part to the following. First, requiring that CLUSTERS strive toward a spherical shape even during development was an unrealistic specification. As evidenced by analogue visualization during execution, CLUSTERS adopt various shapes as they develop. Additionally, CLUSTERS begin as clumps; during subsequent development, they can contain member CELLS inside the enclosed LUMINAL SPACE. Just as not all member CELLS on the grid compose CLUSTER edges, not every off-the-grid CELL will be part of a CLUSTER surface in 3D. Because the number of off-the-grid member CELLS is used to estimate CLUSTER surface area, the computed ratio can be too low. We observed AT II cells in vitro change their size as they aggregated and formed multi-CELL structures. During simulations, all CELLS maintain the same size, and that could contribute to a further deviation of in silico cyst diameters from in vitro data. The finalized implementation used a threshold function that yielded CYST diameters comparable to the experimental data. The threshold is a sigmoidal function of the total number of member CELLS in a CLUSTER:

$$v(i) = \frac{1}{\left(1+e^{-(N_i-a)/b}\right)} \tag{1}$$

where $v(i)$ is the computed threshold of CLUSTER i, N_i is the total number of its member CELLS, and a and b are translation and scale parameters. When a specified axiom applies, the decision-making CELL draws a PRN from a uniform distribution, $U(0,1)$. If the PRN is less than the parent CLUSTER'S computed threshold, the decision-making CELL withdraws itself off the grid; else, it executes an action specified by the applicable axiom. To preclude unintended withdrawal of member CELLS from a small incipient CLUSTER, we defined a minimum number (3), below which the threshold does not apply. CELLS within larger CLUSTERS are more likely to be displaced out of the grid.

Mechanism versus phenomenon within AT II cell cultures

Specification of mechanism and phenomena within a biological system are context, aspect, and perspective dependent. The mechanisms driving higher-level phenomena become phenomena in their own right, driven by even lower level mechanisms. Furthermore, because organismic behaviors can have cascade-down effects that influence events at several levels, including gene expression, higher-level phenomena can become part of (the cause of) the mechanism of lower level events. In that case, the lower level events become the phenomena. So, to what (in the referent system) might in silico operating principles map? We suggest that the answer depends on aspects of AT II cultures under study, the intended model use, and the perspective through which the systems are studied. For this report, the prominent perspective is that of the time-lapse videos depicting cellular and morphological aspects of ALC formation. From the recorded observations, we specified basic, cell-level attributes and behaviors, and created a set of biologically reasonable cell decision logic and axioms that gave rise to the targeted cell mimetic behaviors and developmental patterns. In that respect, we can consider the axiomatic principles of operation and their implementation as (albeit abstract) mechanisms that manifest as cellular behaviors (phenomena), which, in turn, drive the higher-level phenomena of ALC formation. For the same reason, one may view the operating principles as logic descriptions of cell-level phenomena that arise from the workings of lower-level physicochemical mechanisms.

Analogue and in vitro experimental measures

We manually counted the number of AT II clusters formed in time-lapse images obtained after 135 h of culture. In sparse cultures, clusters clearly separated. That enabled accurate total counts. However, in cultures having higher cell densities, cells tended to form clusters in close proximity to one another and we found it challenging to visually separate one cluster from two or more adjacent clusters. In those circumstances, we counted conservatively. Consequently, it is likely that our measure somewhat underestimated the real cluster count at higher cell densities. For other in vitro measures, refer to [9].

During and after termination of simulations, we measured the number of CLUSTERS formed and their sizes, the number of CYSTS and ALC and their sizes, migration distances of individual CELLS and CLUSTERS, and the usage frequency of CELL actions and axioms. Measurements were recorded every simulation cycle. CLUSTER size was measured by counting its member CELLS on and off grid. Every CLUSTER maintained records of its on-the-grid and off-the-grid member CELLS as well as the enclosed LUMINAL SPACE objects. Measurement of the number of CYSTS and their sizes was similar. A non-empty CLUSTER with at least one member LUMINAL SPACE constituted a CYST. The number of CYSTS in a CULTURE corresponded to the number of CLUSTERS having at least one LUMINAL SPACE object and enclosing CELLS. CYST size was quantified using different methods. CYST size can be expressed as the number of member CELLS, or the number of enclosed LUMINAL SPACE objects, or a combination. An alternate method was to compute actual CYST diameters in multiple directions and average them. Following experimentation with various methods, we elected to use the number of enclosed LUMINAL SPACE objects to estimate diameter. The estimation function was:

$$d(c) = 2 + 2\sqrt{L_c / \pi} \tag{2}$$

where $d(c)$ is the computed diameter of CYST c, and L_c is the number of the enclosed LUMINAL SPACE objects. The smallest possible CYST comprised one LUMINAL SPACE object and six enclosing CELLS, so the minimum diameter computes to ~ 3.1 grid units. The method assumed that the CYST had a spherical shape (when mapped to continuous space) and its LUMINAL SPACE was enclosed by a monolayer of CELLS. The estimation became less accurate when the CYST had an irregular shape or multiple layers of CELLS enclosing the LUMINAL SPACE.

The cumulative migration distances of individual CELLS were measured each simulation cycle. To simplify implementation, every CELL maintained its own record of total migration length from the start of a simulation. The cumulative usage of individual CELL actions or axioms was also updated each simulation cycle. When a CELL selected an axiom and executed an action, it invoked the OBSERVER to increment the counter corresponding to that choice and action. The OBSERVER maintained a record of CELL action and axiom usage. Execution counts of CELL migration and CELL-CELL attachments were recorded. At simulation's end, the OBSERVER record was transferred to a data file created for each simulation run.

Simulating AT II cell cultures having altered properties

We investigated behavioral and phenotypic consequences of altering CELL migration mode, CELL-CELL attachment probabilities, CELL migration speed, and the axioms governing clustered CELL actions.

Every CELL or CLUSTER has a parameter that dictates its mode of migration. CULTURE has a global parameter to set the initial migration mode. We explored the three migration modes in standard simulation experiments using varying initial CELL populations. Collectively, the experiments comprised 18,000 Monte Carlo (MC) runs: 3 movement modes x 60 initial CELL densities x 100 repeat simulations. We also explored a hybrid migration mode; it enabled a CELL to switch from CELL density-based movement mode to random movement with a certain probability. The probability of random movement ranged from 0 to 1 in increments of 0.1.

We varied CELL-CELL attachment probabilities parametrically and studied how the changes affected CULTURE phenotype. One parameter having a Table 2 value of 0.2 controlled the probability of CELL-CELL attachment initiated by a non-clustered CELL. A separate parameter having a low Table 2 value of 0.01 specified the probability of attachment initiated by a clustered CELL. We used eleven different probability values from zero to one, in increments of 0.1, for each of the two attachment parameters. The experiments collectively comprised to 2,178,000 individual MC runs: 11 x 11 (values of two probability parameters) x 3 movement modes x 60 initial CELL densities x 100 repeat simulations.

We simulated migration speed changes by separately adjusting CELL and CLUSTER speeds parametrically. The Table 2 parameter value was one grid unit per simulation cycle. Separate parameters were used to change the speeds of CELLS and CLUSTERS. We explored ten different speeds for each, from zero to two grid units per simulation cycle in increments of 0.2 grid units per cycle. All three CELL migration modes were tested. The simulation experiments collectively comprised 1,800,000 individual MC runs: 10 x 10 (values of the two speed parameters) x 3 movement modes x 60 initial CELL densities x 100 repeat simulations.

To investigate the roles and relative importance of clustered CELL axiomatic operating principles (Fig. 4) in ALC formation, each axiom was muted separately and its effect on CULTURE phenotype catalogued. Each axiom was muted by changing its action to do nothing. So doing provided basic, limited insight, which was sufficient for the aims of this study but should be explored further in future rounds of analogue revision. The AT II analogue used 13 axioms. Axioms 1b, 2c, 2g, and 3c specify do nothing, so they were excluded from the analysis. The remaining axioms dictated actions that changed the location of the decision-making CELL. A simulation experiment corresponded to silencing of one axiom. All parameters and operations used the Table 2 values. The analogue's behavior was studied in the visual mode. Each simulation experiment was repeated ten times; 90 simulations were examined.

References

1. Grimm V, Revilla E, Berger U, Jeltsch F, Mooij WM, Railsback SF, Thulke HH, Weiner J, Wiegand T, DeAngelis DL (2005) Pattern-oriented modeling of agent-based complex systems: lessons from ecology. *Science* 310: 987–991.
2. Law AM, Kelton WD (2000) Simulation modeling and analysis. New York: McGraw-Hill. 784 p.
3. Zeigler BP, Kim TG, Praehofer H (2000) Theory of modeling and simulation: integrating discrete event and continuous complex dynamic systems. San Diego: Academic Press. 510 p.
4. Kiczales G, Lamping J, Mendhekar A, Maeda C, Lopes C, Loingtier J-M, Irwin J (1997) Aspect-oriented programming. *Proceedings of the European Conference on Object-Oriented Programming* 1241: 220–242.
5. Friedl P, Hegerfeldt Y, Tusch M (2004) Collective cell migration in morphogenesis and cancer. *Int J Dev Biol* 48: 441–449.
6. Kolega J (1981) The movement of cell clusters in vitro: morphology and directionality. *J Cell Sci* 49: 15–32.
7. Pankov R, Endo Y, Even-Ram S, Araki M, Clark K, Cukierman E, Matsumoto K, Yamada KM (2005) A Rac switch regulates random versus directionally persistent cell migration. *Cell Biol* 170: 793–802.
8. Lesur O, Arsalane K, Lane D (1996) Lung alveolar epithelial cell migration in vitro: modulator and regulation processes. *Am J Physiol* 270: L311–L319.
9. Yu W, Fang X, Ewald A, Wong K, Hunt CA, Werb Z, Matthay MA, Mostov K (2007) Formation of cysts by alveolar type II cells in three-dimensional culture reveals a novel mechanism for epithelial morphogenesis. *Mol Biol Cell* 18: 1693–1700.
10. Kheradmand F, Folkesson HG, Shum L, Derynk R, Pytela R, and Matthay MA (1994) Transforming growth factor-γ enhances alveolar epithelial cell repair in a new in vitro model. *Am J Physiol* 267: L728–L738.
11. Lo CM, Wang HB, Dembo M, Wang YL (2000) Cell movement is guided by the rigidity of the substrate. *Biophys J* 79: 144–152.
12. Even-Ram S, Yamada KM (2005) Cell migration in 3D matrix. *Curr Opin Cell Biol* 17: 524–532.

13. O'Brien LE, Zegers MM, Mostov KE (2002) Building epithelial architecture: insights from three-dimensional culture models. *Nat Rev Mol Cell Biol* 3: 531–537.

14. Gibson MC, Patel AB, Nagpal R, Perrimon N (2006) The emergence of geometric order in proliferating metazoan epithelia. *Nature* 442: 1038–1041.

15. Grant MR, Mostov KE, Tlsty TD, Hunt CA (2006) Simulating properties of in vitro epithelial cell morphogenesis. *PLoS Comput Biol* 2: e129.
