# Supplementary figures and images for: A Computational Approach to Understand In Vitro Alveolar Morphogenesis
Source: PLoS One. 2009 Mar 13;4(3):e4819. doi: 10.1371/journal.pone.0004819 (PMC2653231; doi:10.1371/journal.pone.0004819)

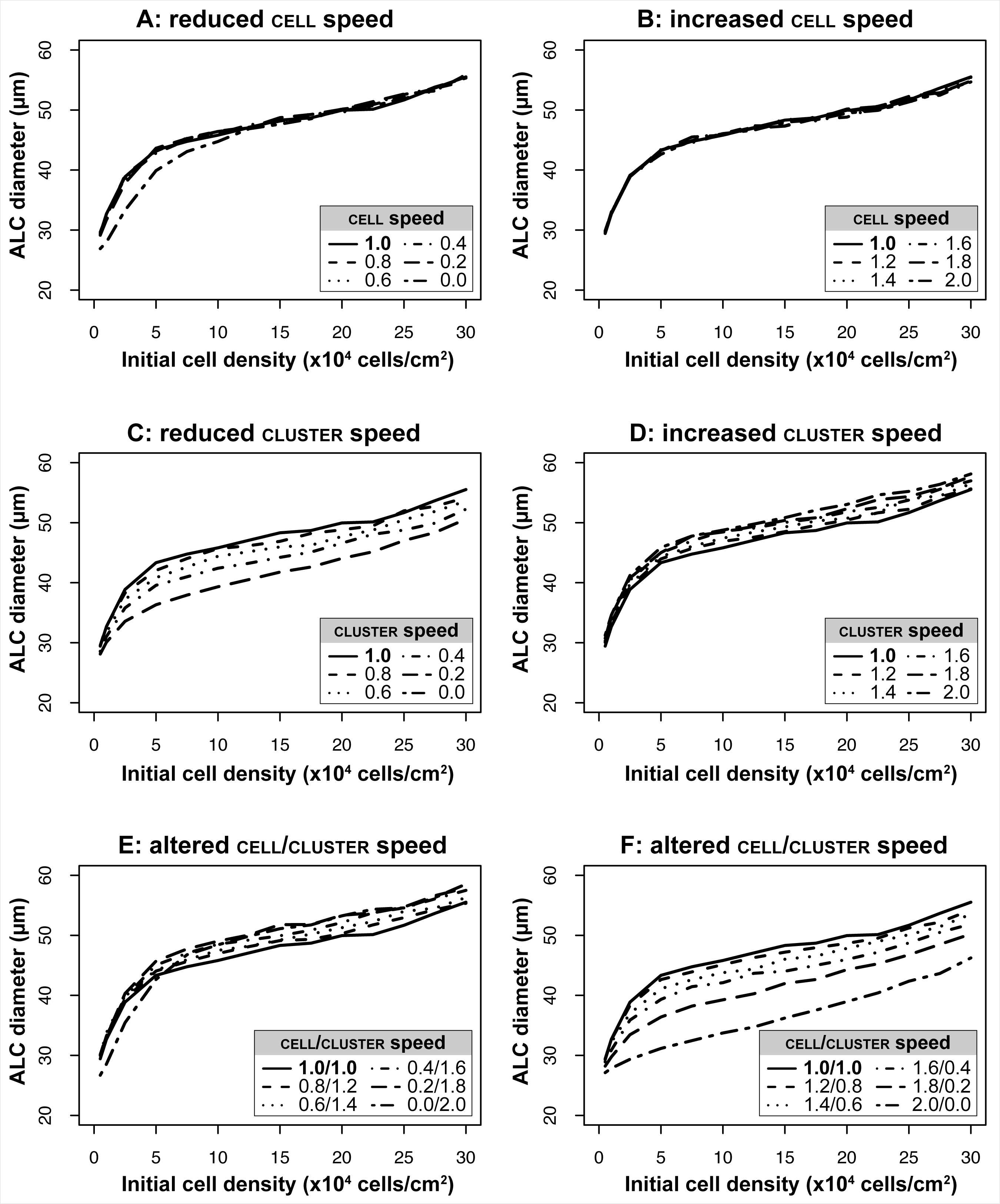

Supplement: Figure S1 — Altered alveolar-like cyst (ALC) growth in silico following changes in simulated cell and cluster speed (chemotactic mode). Single and collective cell migration speeds are controlled parametrically. Cluster migration implements collective cell migration. Individual cells and clusters were directed to migrate chemotactically along a local cell-produced attractant gradient. All other model parameters were set to the Table 2 values. Increasing or decreasing cell speed had a material effect on ALC growth. (A) Reduced single cell speed; (B) increased single cell speed; (C) reduced cluster speed; (D) increased cluster speed; (E–F) simultaneous reduction/increase in single/collective cell speed. We executed 100 Monte Carlo runs per cell density; each run lasted 100 simulation cycles. (8.80 MB TIF) [file pone.0004819.s002.tif]

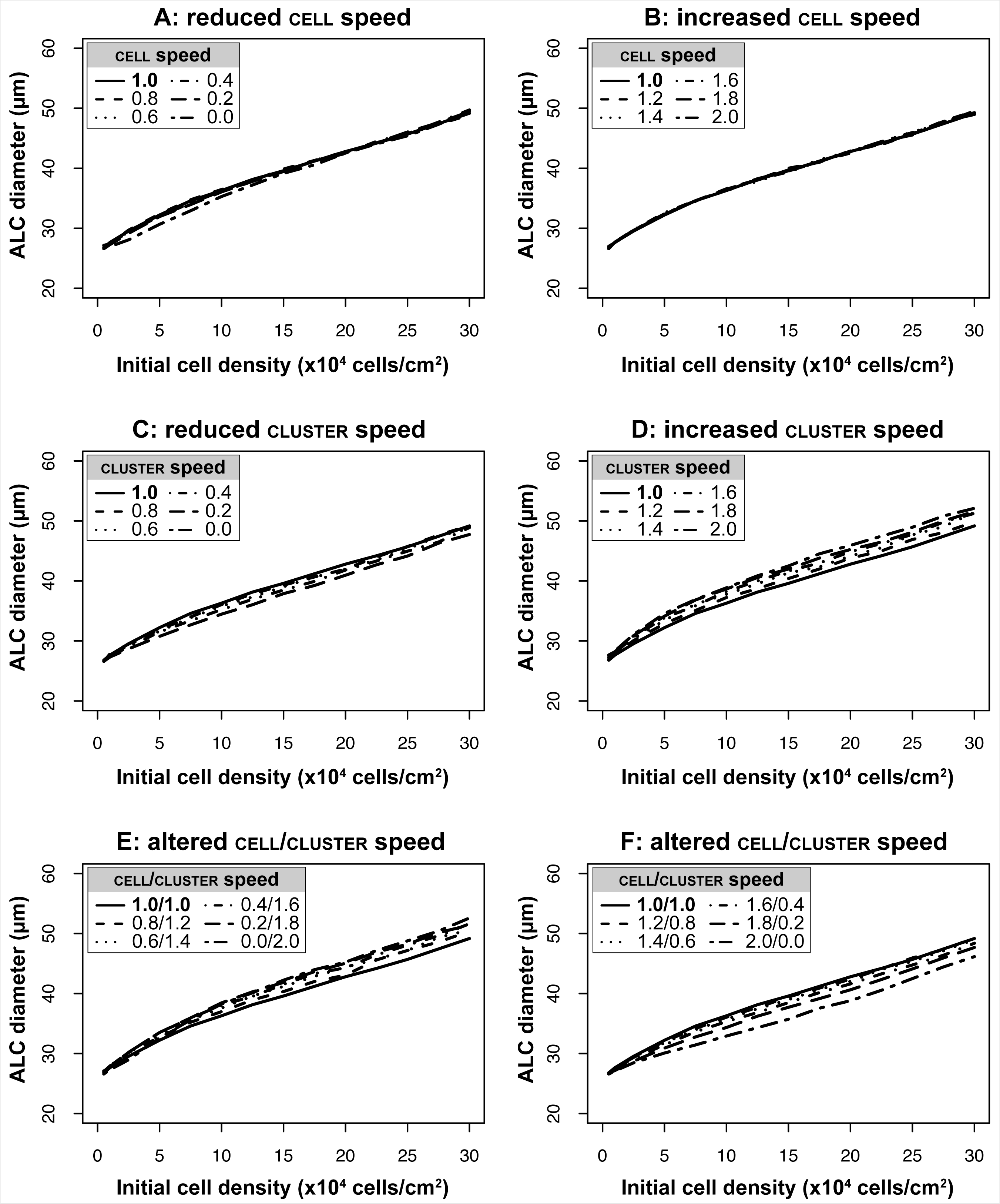

Supplement: Figure S2 — Altered ALC growth in silico following changes in simulated cell and cluster speed (random migration mode). The experiments are the same as those in Fig. S1 except for the migration mode used. (8.82 MB TIF) [file pone.0004819.s003.tif]
